# Beyond combination: sequencing priming modalities before immune checkpoint inhibitors to convert immunologically cold tumors toward an ICI-responsive phenotype

**DOI:** 10.3389/fimmu.2026.1947737

**Published:** 2026-10-05

**Authors:** Weiqiang Fan, Hui Xu, Yating Wu, Yonghai Peng

**Affiliations:** Department of Oncology, The Cangshan District of the 900th Hospital, Fuzhou, Fujian, China

**Keywords:** cold tumors, immune checkpoint inhibitors, radiotherapy, treatment sequencing, tumor microenvironment

## Abstract

Immune checkpoint inhibitors have transformed oncology but largely spare immunologically “cold” tumors, those with sparse T-cell infiltration, low PD-L1 expression, and an immunosuppressive microenvironment. The prevailing response has been a combination: adding a priming modality to a checkpoint inhibitor, most often concurrently. We argue that this “addition” paradigm has plateaued in cold disease because it treats combination as arithmetic rather than as a temporally ordered program, and that timing, the order and interval of interventions, may govern outcome as much as the choice of agents. We first define what makes a tumor immunologically cold and why simultaneous stacking reaches a ceiling. We then assemble the clinical evidence that timing already shapes outcomes: most maturely in the scheduling of radiotherapy relative to checkpoint blockade and, strikingly, in neoadjuvant-versus-adjuvant immunotherapy, where the same agent changes outcome according to when it is given. We synthesize the temporal immunobiology that makes ordering matter—antigen release and dendritic-cell priming kinetics, the vascular-normalization window, compensatory adaptive-resistance windows, and the slow dynamics of the gut microbiome—and review, mechanism by mechanism, the timing evidence for microenvironment priming, checkpoint inhibition, and microbiome modulation, emphasizing that mistiming can be harmful rather than merely ineffective. We describe how several groups are translating this principle into integrated, time-sequenced strategies and set out, as one concrete and falsifiable instantiation, a three-axis schema, which we term TMEp-CI-M, illustrated by cross-tumor proof-of-concept experience and framed as hypothesis-generating rather than validated. A therapy built on timing will require dynamic biomarkers of window state and adaptive, biomarker-driven trials. For cold tumors, re-sequencing the agents we already have, rather than adding new ones, may be among the highest-yield and lowest-cost hypotheses in immuno-oncology and one that can be tested now.

## 1 Introduction

Immune checkpoint inhibitors (ICIs) have transformed the treatment of many malignancies (1), yet their benefit remains concentrated in tumors that are already immunologically “hot.” A large fraction of solid cancers present instead as immune-excluded or immune-desert lesions, marked by sparse CD8^+^ T-cell infiltration, low PD-L1 expression, and a suppressive stroma; these “cold” tumors respond poorly to checkpoint blockade alone (2). The clinical footprint of this problem is broad. In metastatic non-small cell lung cancer (NSCLC), roughly half of the patients are PD-L1-negative (3), and even when an ICI is added to chemotherapy the incremental survival gain in that subgroup narrows over time (4). In advanced hepatocellular carcinoma (HCC), single-agent PD-1 blockade failed to improve overall survival over sorafenib in CheckMate 459 (5) and did not meet its endpoint as second-line therapy in KEYNOTE-240 (6). The same pattern holds across the coldest histologies: pancreatic ductal adenocarcinoma, mismatch-repair-proficient colorectal cancer, and most small-cell and neuroendocrine carcinomas derive little or no benefit from checkpoint blockade alone (7–9) and together account for a substantial share of cancer mortality. Converting cold tumors into checkpoint-responsive disease is, therefore, among the most consequential unmet needs in clinical oncology.

The dominant response has been combination: stacking a priming modality (radiotherapy, anti-angiogenics, chemotherapy, or microbiome intervention) onto an ICI, most often concurrently. This “addition” logic has produced genuinely practice-changing regimens, from atezolizumab plus bevacizumab in HCC (10) to numerous PD-1/tyrosine-kinase-inhibitor pairings (11, 12) and dual checkpoint blockade in melanoma (13). Yet in genuinely cold disease, combination has not reliably delivered: concurrent stacking adds toxicity (14, 15) [in the randomized KEYNOTE-A18 trial, for example, concurrent pembrolizumab with chemoradiotherapy roughly doubled immune-mediated adverse events, 39% versus 17% (15)], is usually assembled around what to give rather than when each agent should act, and still leaves a large non-responding fraction. The implicit premise (that giving more, together, is better) is increasingly hard to reconcile with the underlying immunobiology.

A different principle is emerging from the data: for cold tumors, timing, the order and interval of interventions, may govern outcome as much as the choice of agents. The clearest signal comes from radiotherapy–ICI combinations. A recent network meta-analysis of 24 randomized trials (9,480 patients) found that sequential ICI administered after radiotherapy improved overall and progression-free survival, whereas concurrent radiotherapy plus ICI did not achieve the same benefit (16); the PACIFIC/COAST consolidation paradigm operationalizes the same prime-then-inhibit sequence at a practice-changing scale (17, 18). The corollary, that mistiming can actively harm, is equally instructive: aggressive antibiotic pre-conditioning immediately before ICI disrupted the microbiome and impaired systemic immunity in a randomized melanoma trial (19). Converting a cold tumor, in other words, is not a single switch but a temporally ordered program, each step of which addresses a different missing element of the cancer-immunity cycle (20).

Despite these converging threads, the field still discusses the sequencing question one modality at a time, most directly for radiotherapy and ICIs (21), and microenvironment priming, checkpoint inhibition, and microbiome modulation have rarely been considered together along a single time axis. This review is organized around that gap. We first define what makes a tumor immunologically cold and why single interventions and simultaneous addition reach a ceiling (Sections 2 to 3). We then assemble the empirical case that timing already shapes outcomes (Section 4) and develop the temporal immunobiology that makes ordering matter (Section 5). We review, mechanism by mechanism, the timing evidence for microenvironment priming, checkpoint deployment, and microbiome conditioning (Sections 6–8), together with the dynamic biomarkers any timing-based strategy will require (Section 9). Finally, we consider how these strands are being integrated into time-sequenced strategies, describe one concrete, falsifiable instantiation (Section 10), and set out the open questions that will decide whether, for cold tumors, the order of interventions is itself a determinant of benefit (Section 11). We treat sequencing throughout not as an alternative to combination but as its necessary discipline.

## 2 The biology of the cold tumor: why single interventions fail

Immune “coldness” is a composite phenotype rather than a single deficiency. In the widely used framework of Galon and Bruni, tumors are stratified as immune-inflamed (“hot”), immune-altered (excluded or immunosuppressed), and immune-desert (“cold”), reflecting the density, location, and functional state of tumor-infiltrating lymphocytes (2). The functional core of this distinction is the presence of pre-existing, tumor-reactive CD8^+^ T cells: in melanoma, a dense CD8^+^ infiltrate at the invasive margin is the strongest single correlate of response to PD-1 blockade, and its relative absence is, in effect, the operational definition of a cold tumor (22). Coldness is, thus, better understood not as one deficiency but as a low “cancer-immune set point”—the integrated output of tumor, host, and environmental factors that determines whether an antitumor response can be mounted at all (23). Cold tumors combine several failing axes: scant intratumoral CD8^+^ T cells, low or absent PD-L1 expression, low tumor mutational burden (TMB), a muted interferon-γ (IFN-γ) transcriptional signature, and a stroma enriched for regulatory T cells and myeloid-derived suppressor cells (2).

Several distinct mechanisms drive the cold state, and their diversity maps onto the now-standard taxonomy of primary, adaptive, and acquired resistance to immunotherapy (24, 25)—and is itself an argument for multi-mechanism therapy. First, tumor-cell-intrinsic oncogenic signaling can exclude T cells outright: activation of the WNT/β-catenin pathway suppresses the chemokines needed for dendritic-cell recruitment and produces a non-T-cell-inflamed microenvironment that resists anti-PD-1/anti-CTLA-4 therapy (26). Second, even when tumor-reactive T cells exist, the stroma can physically exclude them from cancer-cell nests, a form of immune privilege imposed by the microenvironment rather than by the T cell itself (27). Third, defects in the IFN-sensing and antigen-presentation machinery blunt the response even when T cells reach their target: loss-of-function mutations in JAK1/JAK2 or in B2M underlie acquired resistance to PD-1 blockade by rendering tumor cells insensitive to IFN-γ or unable to display antigen (28). Fourth, the quantity and quality of neoantigens set an upper bound on what checkpoint release can achieve. The antigens that checkpoint blockade actually mobilizes are predominantly tumor-specific mutant proteins (29), so higher TMB is associated with improved response and survival after ICI across diverse histologies, albeit with tumor-type-specific thresholds (30). The relationship was first established in NSCLC, where nonsynonymous mutation burden predicted response, durable benefit, and progression-free survival on pembrolizumab (31). Crucially, it is the clonal fraction that matters: tumors enriched for clonal neoantigens, carried by every cancer cell, are far more sensitive to PD-1 and CTLA-4 blockade than tumors in which the same mutational load is dispersed subclonally (32). Pan-tumor analyses identify clonal TMB together with T-cell-recruiting chemokine programs (e.g., CXCL9) as the strongest determinants of checkpoint sensitivity (33).

Two conclusions follow. First, a cold tumor typically fails on more than one of these axes at once, so single-agent or single-mechanism approaches address only part of the deficit and predictably stall. Second, because the antitumor response is itself a cyclical, ordered process—antigen release, presentation, priming, trafficking, infiltration, recognition, killing (20)—”warming” a tumor is less a matter of pushing one lever harder than of restoring several missing steps in sequence. This multi-axis, multi-step phenotype recurs across histologies that share little else molecularly—pancreatic ductal adenocarcinoma, mismatch-repair-proficient colorectal cancer, many hepatocellular and small-cell lung cancers, and neuroendocrine tumors—which is why a mechanism-based, rather than tumor-type-based, strategy for warming them is attractive and why the ordering of interventions, not merely their selection, becomes the central design question.

The way in which tumors are cold, however, differs by histology, and this shapes how each might be warmed. Pancreatic ductal adenocarcinoma is walled off by a dense, fibrotic, hypoperfused stroma that both excludes T cells and compresses tumor vasculature—an exclusion phenotype in which relieving the physical and vascular barrier is a prerequisite for any checkpoint effect (27). Glioblastoma combines an immune-sequestered, low-perfusion site with profound myeloid-driven immunosuppression and systemic T-cell dysfunction. Mismatch-repair-proficient colorectal cancer is antigen-poor and non-inflamed despite an intact host immune system, whereas small-cell lung cancer and many neuroendocrine carcinomas are highly proliferative yet immunologically near-silent. These are not variations on a single defect but distinct combinations of the axes above, which is why a single generic primer is unlikely to suffice and why the choice and ordering of the priming modality may need to be matched to the dominant barrier in each disease. **Table 1** sets out, for each of these histologies, the barrier that predominates, what that barrier does to the priming step, and the adjustment to sequence it implies.

**Table 1:** How cold tumors are cold: the dominant barrier by histology and the sequencing adjustment it implies.

Tumor type	Dominant barrier to T-cell engagement	Practical consequence for the priming step	Sequencing adjustment this implies	Ref.
Pancreatic ductal adenocarcinoma	Dense fibrotic stroma with vascular compression and hypoperfusion, on an antigen-poor background	A primer cannot perfuse the target while the physical and vascular barrier stands, so antigen released at the rim never reaches a functioning vessel	Relieve the vascular and stromal barrier first, then prime, then release the checkpoint; one generic primer is unlikely to suffice	(27, 58, 75)
Glioblastoma	Immune-sequestered, low-perfusion site with myeloid-driven suppression and systemic T-cell dysfunction	Systemic priming reaches the lesion poorly, and the circulating T-cell pool that a checkpoint inhibitor must act on is itself depleted	Give checkpoint exposure before resection, while the antigen source and its draining nodes are still intact	(38, 39)
Mismatch-repair-proficient (microsatellite-stable) colorectal cancer	Antigen-poor and non-inflamed despite intact host immunity	There is little to release: blockade acts on a repertoire that has not yet been generated	An antigen-generating primer must precede the inhibitor; concurrent stacking adds toxicity without adding antigen	(2, 77, 111)
PD-L1-negative non-small-cell lung cancer	Sparse CD8 infiltrate at the invasive margin with absent target expression	Single-agent benefit is small because the infiltrate has to be induced rather than disinhibited	Radiotherapy first, then PD-1 blockade within the post-radiation interval	(3, 35, 56, 110)
Hepatocellular carcinoma	Abnormal, hypoxia-driven vasculature inside a constitutively tolerogenic organ	Perfusion rather than antigen is usually the rate-limiting step	Low-dose anti-angiogenic conditioning to open the normalization window, with blockade timed inside it	(10, 41, 78, 115)
Small-cell lung cancer and neuroendocrine carcinoma	Highly proliferative yet immunologically near-silent, with a fast competing risk of progression	The window has to be opened quickly, and disease may outrun any long lead-in	Short-interval or overlapping schedules, where the competing risk legitimately favors compression	(9, 109, 114)

Coldness is a composite phenotype, and the axis on which a tumor fails differs by histology. For each disease, the table gives the barrier that predominates, what that barrier does to the priming step, and the consequence for the order in which agents are given. The final column is a hypothesis generated by the framework of this review rather than a validated recommendation. The supporting references are of mixed level, ranging from randomized trials to single-arm series and preclinical work. None of these adjustments has been tested in a randomized comparison of schedule.

CD8, cluster of differentiation 8; PD-1, programmed cell death protein 1; PD-L1, programmed death-ligand 1.

## 3 From combination to sequence: the limits of concurrent addition

Confronted with checkpoint resistance, the field has largely responded by combination—adding a second modality to an ICI, most often on a concurrent schedule. In hot and intermediate disease, this has been transformative: atezolizumab plus bevacizumab became first-line standard in HCC (10), analogous PD-1/anti-angiogenic pairings such as sintilimab plus a bevacizumab biosimilar and camrelizumab plus apatinib extended the template—the latter achieving objective response rates of 34% in the first-line and 23% in the second-line setting (11, 12)—and dual PD-1/CTLA-4 blockade delivered durable long-term survival in advanced melanoma (13). The conceptual momentum behind ever-broader combinations is considerable, and the pipeline of checkpoint-based doublets and triplets continues to expand (34). Combination is not the problem; in the right disease, it is the foundation of modern immuno-oncology.

For genuinely cold tumors, however, the addition paradigm has plateaued. In PD-L1-negative NSCLC, which accounts for approximately half of all metastatic NSCLC (3), the survival advantage of adding an ICI to chemotherapy erodes over time (4), and single-agent checkpoint blockade has repeatedly failed to meet its endpoints in cold disease such as advanced HCC (5, 6). Three limitations recur. First, concurrent stacking compounds toxicity without proportionate efficacy gains, a liability that is magnified in the frail, heavily pre-treated patients who typify cold disease. Second, most combinations are assembled around what to give rather than when, with no explicit biological rationale for the interval between priming and checkpoint blockade. Third, in the coldest tumors, a large non-responding fraction persists regardless of how many agents are added simultaneously. Each added agent also carries its own toxicity and cost, so the therapeutic index of stacking narrows even as its incremental efficacy in cold disease flattens—an unfavorable trade that pure combination cannot escape.

The unifying weakness is conceptual. Treating combination as arithmetic addition assumes that the contribution of each agent is fixed and independent of the others’ timing. However, if the antitumor response is a temporally ordered program (Section 2), then the value of each intervention depends on the state that the preceding step has created: a checkpoint inhibitor can only disinhibit T cells that priming has already generated, and a primer wasted before the host can mount any systemic response yields little. From this vantage, the persistent non-responding fraction in cold disease is not necessarily evidence that the right agents are missing—it may be evidence that the right agents are being given in the wrong order or at the wrong interval. If non-response reflects mistiming rather than missing agents, then the same agents, re-ordered, might succeed where their simultaneous combination failed, at no additional drug cost and often at lower toxicity. The remainder of this review develops the argument that the missing dimension is not another agent but the ordering of several, matched to the biology each is meant to repair.

## 4 Timing already shapes outcomes: clinical signals across the field

The proposition that when an immunotherapy is given can change outcome is not speculative; elements of it are already embedded in clinical practice, though almost always for one modality at a time. Assembling these scattered signals is the empirical foundation for a timing-based approach (**Figure 1**).

**Figure 1:**
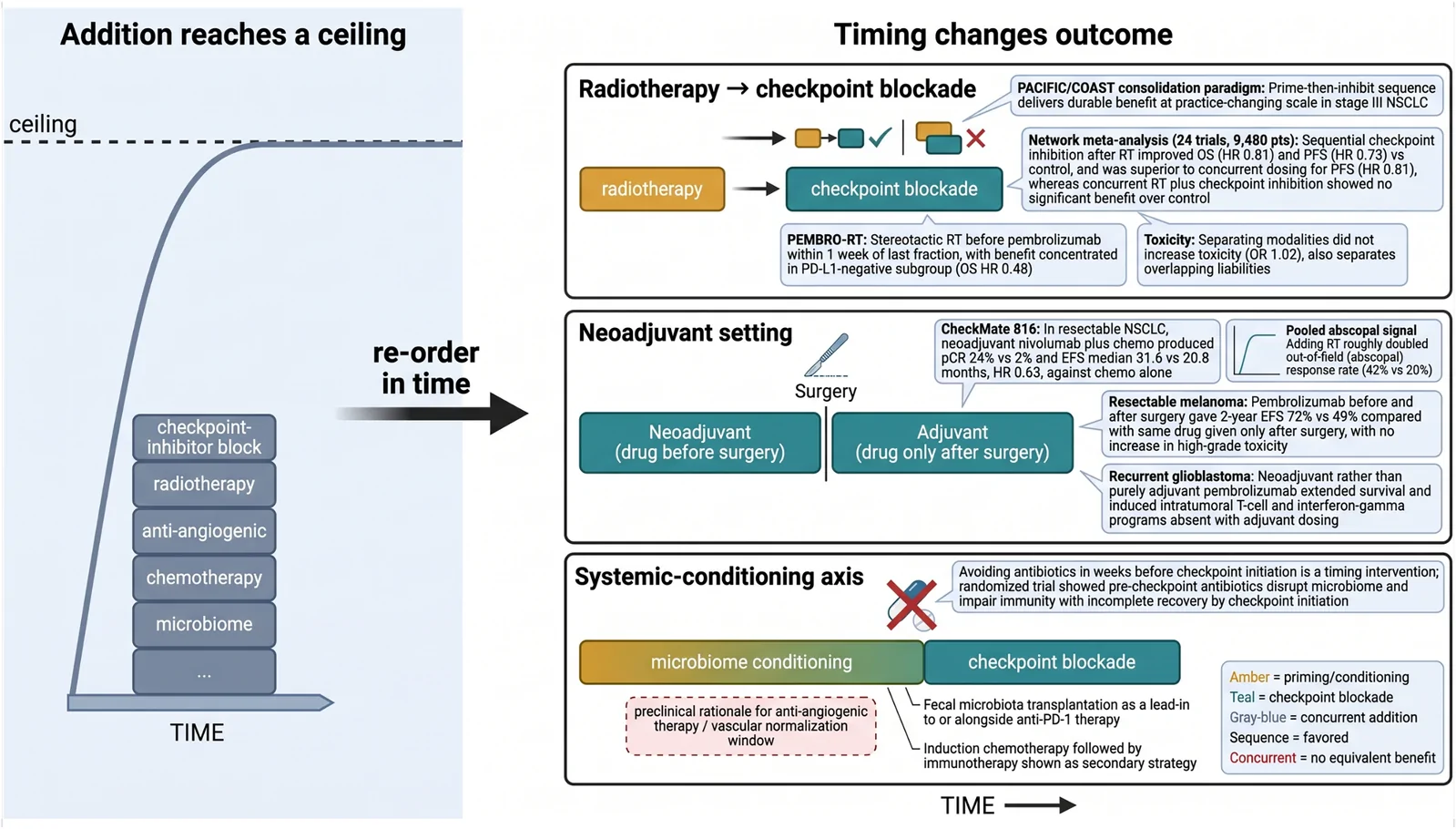
Addition reaches a ceiling, whereas re-ordering the same agents in time changes the outcome. The left panel shows that stacking a checkpoint inhibitor with priming modalities (radiotherapy, anti-angiogenic, chemotherapy, and microbiome) concurrently reaches a benefit ceiling. An arrow (“re-order in time”) leads to three panels that assemble the clinical evidence that a priming or conditioning step delivered before checkpoint blockade tends to outperform the same components given together or in reverse order. Radiotherapy then checkpoint blockade: the PACIFIC/COAST consolidation paradigm is a prime-then-inhibit sequence delivering durable benefit at a practice-changing scale in stage III non-small cell lung cancer (17, 18); a network meta-analysis (24 trials, 9,480 patients) found sequential checkpoint inhibition after radiotherapy improved overall survival (hazard ratio 0.81) and progression-free survival (hazard ratio 0.73) versus control and was superior to concurrent dosing for progression-free survival (hazard ratio 0.81), whereas concurrent radiotherapy plus checkpoint inhibition showed no significant benefit over control (16); PEMBRO-RT delivered stereotactic radiotherapy before pembrolizumab within 1 week of the last fraction, with benefit concentrated in the PD-L1-negative subgroup (overall-survival hazard ratio 0.48) (35); separating the modalities in time did not increase toxicity (odds ratio 1.02) and also separates overlapping liabilities (16). Neoadjuvant setting, where the same agent is moved relative to a surgery landmark: neoadjuvant nivolumab plus chemotherapy versus chemotherapy alone produced pathological complete response of 24% versus 2% and event-free survival of 31.6 versus 20.8 months (hazard ratio 0.63) in resectable non-small cell lung cancer (CheckMate 816) (36); peri-operative pembrolizumab given before and after surgery gave 2-year event-free survival of 72% versus 49% compared with the same drug given only after surgery, with no increase in high-grade toxicity, in resectable melanoma (37); and in recurrent glioblastoma, neoadjuvant rather than purely adjuvant pembrolizumab extended survival and induced intratumoral T-cell and interferon-γ programs absent with adjuvant dosing (38). A pooled analysis showed that adding radiotherapy roughly doubled the out-of-field (abscopal) response rate (42% versus 20%) (56). Systemic-conditioning axis: microbiome conditioning begins before and continues alongside checkpoint blockade; fecal microbiota transplantation is given as a lead-in to or alongside anti-PD-1 therapy, avoiding antibiotics in the weeks before checkpoint initiation is itself a timing intervention because pre-checkpoint antibiotics disrupt the microbiome and impair immunity (19), and induction chemotherapy followed by immunotherapy embodies the same logic; the anti-angiogenic vascular-normalization window is shown with a dashed outline to denote preclinical rationale. Amber denotes priming or conditioning, teal denotes checkpoint blockade, and gray-blue denotes concurrent addition. All values are as reported in the cited trials; the sequential-versus-concurrent comparison rests on a network meta-analysis rather than a head-to-head sequencing trial. Created in https://BioRender.com.

The most mature strand is the scheduling of radiotherapy relative to checkpoint blockade. Consolidation immunotherapy after completion of chemoradiotherapy, the PACIFIC paradigm and its platform successor COAST, is a prime-then-inhibit sequence by construction, and it delivered a durable survival benefit at a practice-changing scale in stage III NSCLC (17, 18). A network meta-analysis of 24 randomized trials found that ICI given sequentially after radiotherapy outperformed concurrent co-administration on progression-free survival and improved survival relative to control where concurrent dosing did not (16), while trials such as PEMBRO-RT deliberately delivered stereotactic radiotherapy before pembrolizumab (35). Dedicated reviews now catalogue the timing, efficacy, and safety data for the radiotherapy–ICI pairing specifically (21). Across these data, the pattern is consistent: radiotherapy delivered as a discrete priming course, with checkpoint blockade following, tends to outperform the two given strictly simultaneously.

The cleanest demonstration that timing *per se* matters, however, comes from the neoadjuvant setting, where the same agent is delivered at different points relative to definitive therapy. In resectable NSCLC, neoadjuvant nivolumab plus chemotherapy produced markedly higher pathological complete-response rates (24% vs. 2%) and longer event-free survival (median 31.6 vs. 20.8 months; hazard ratio 0.63) than chemotherapy alone (CheckMate 816) (36). In resectable melanoma, a randomized trial found that pembrolizumab given both before and after surgery significantly prolonged event-free survival compared with the identical drug given only after surgery—a 2-year event-free survival of 72% versus 49%, with no increase in high-grade toxicity (37). Most strikingly for the cold-tumor argument, a randomized trial in recurrent glioblastoma, an archetypally cold malignancy, found that neoadjuvant, as opposed to purely adjuvant, pembrolizumab significantly extended survival and induced intratumoral T-cell and interferon-γ programs absent with adjuvant dosing (38). When the agent is identical and only its position in time differs, the result is as clean a demonstration as exists that timing can govern outcome; the biological and trial-design implications of this shift have been articulated as a distinct paradigm (39).

Timing is emerging as a variable in the systemic-conditioning axis as well. Fecal microbiota transplantation is now given as a lead-in to, or alongside, anti-PD-1 therapy (40), and the avoidance of antibiotics in the weeks before checkpoint initiation has itself become a timing intervention after a randomized trial showed that pre-checkpoint antibiotics disrupt the microbiome and impair immunity (19). Induction chemotherapy followed by immunotherapy, long used empirically to debulk and prime disease before an ICI is introduced, embodies the same intuition in yet another modality. Even anti-angiogenic therapy, whose vascular “normalization window” is compelling in preclinical models (41), is increasingly discussed in temporal terms (42, 43), although, as Section 6 details, the pivotal trials have not yet exploited that window.

What these strands share is instructive in two ways. First, each independently points in the same direction: a priming or conditioning step, delivered before checkpoint blockade, tends to outperform the same components given together or in the reverse order. Second, each has been developed in a silo defined by the modality rather than by the underlying immunobiology, so the timing of radiotherapy, of surgery-linked immunotherapy, and of microbiome intervention has never been considered on a common axis. The general importance of temporal dynamics across immunotherapy modalities has recently been articulated conceptually (44); what remains missing is an integrative, mechanism-anchored treatment specific to cold tumors. To build toward it, the next section turns from the clinical signal to the biology that explains it.

## 5 The temporal immunobiology: windows of opportunity

If addition has a ceiling and sequence a signal, the reason lies in an immunobiology that is intrinsically time-structured. Four overlapping “clocks” govern whether a cold tumor can be ignited and then exploited; a timing-based strategy is, in essence, an attempt to align interventions to these clocks rather than to the calendar of drug administration (**Figure 2**). **Figure 3** summarizes the cellular and immunological basis of this argument. It first establishes the cold baseline, in which the limiting defect sits upstream of the checkpoint, and then sets out the two orderings that recur throughout Sections 3 to 5. The first is why priming before checkpoint blockade is mechanistically preferable to giving both agents at once. The second is why moving the same checkpoint inhibitor before rather than after definitive surgery changes what the drug can do.

**Figure 2:**
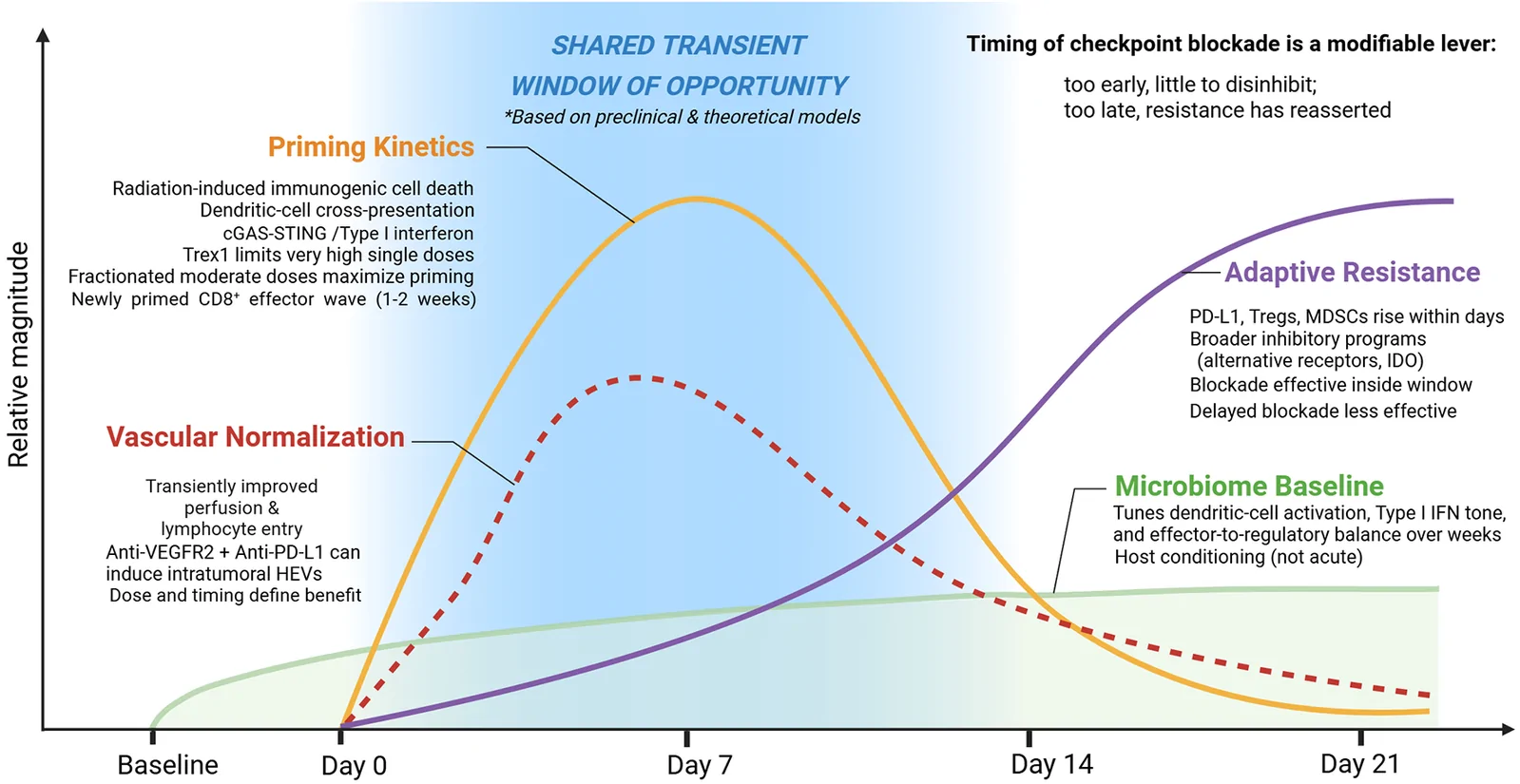
The temporal immunobiology of the cold tumor: four overlapping clocks and a shared transient window of opportunity. The horizontal axis is time in days from the start of priming, with day 0 set at the first radiotherapy fraction or the first dose of whichever priming modality is used, and tick marks at baseline, day 0, day 7, day 14, and day 21. The vertical axis is relative magnitude in arbitrary units; each curve is scaled to its own maximum so that heights are comparable within a curve over time but not between curves, and the axis is, therefore, drawn without numerical tick labels by design. A shaded band, labelled “shared transient window of opportunity”, spans day 1 to day 14, with a particular focus centered around day 7. The image subtitle states the governing principle: the timing of checkpoint blockade is a modifiable lever—too early, and there is little to disinhibit; too late, and resistance has reasserted. Priming kinetics (top left): radiation-induced immunogenic cell death, dendritic-cell cross-presentation, and cGAS–STING/type I interferon signaling culminate in the newly primed CD8+ effector wave, which emerges roughly 1 to 2 weeks after irradiation; the panel notes that the exonuclease Trex1 limits very high single doses, whereas fractionated moderate doses maximize priming. Vascular normalization (bottom left): perfusion and lymphocyte entry improve transiently; anti-VEGFR2 plus anti-PD-L1 can induce intratumoral high endothelial venules, and dose and timing define benefit. The descending limb of this curve reflects loss of normalization through vessel over-pruning under continued or higher-dose exposure and reversion of perfusion once the agent is withdrawn; it is not a depiction of adaptive immune resistance. Adaptive resistance (top right): PD-L1, regulatory T cells, and myeloid-derived suppressor cells rise within days, followed by broader inhibitory programs (alternative receptors, IDO); blockade is effective inside the window and delayed blockade is less effective, which sets the window’s upper bound. Microbiome baseline (bottom right): host conditioning tunes dendritic-cell activation, type I interferon tone, and the effector-to-regulatory balance over weeks; it is a slow baseline, deliberately not acute and not bounded by the shared window. The window is a synthesis of preclinical and theoretical kinetics, not a measured human schedule, as the image footnote states. Created in https://BioRender.com.

**Figure 3:**
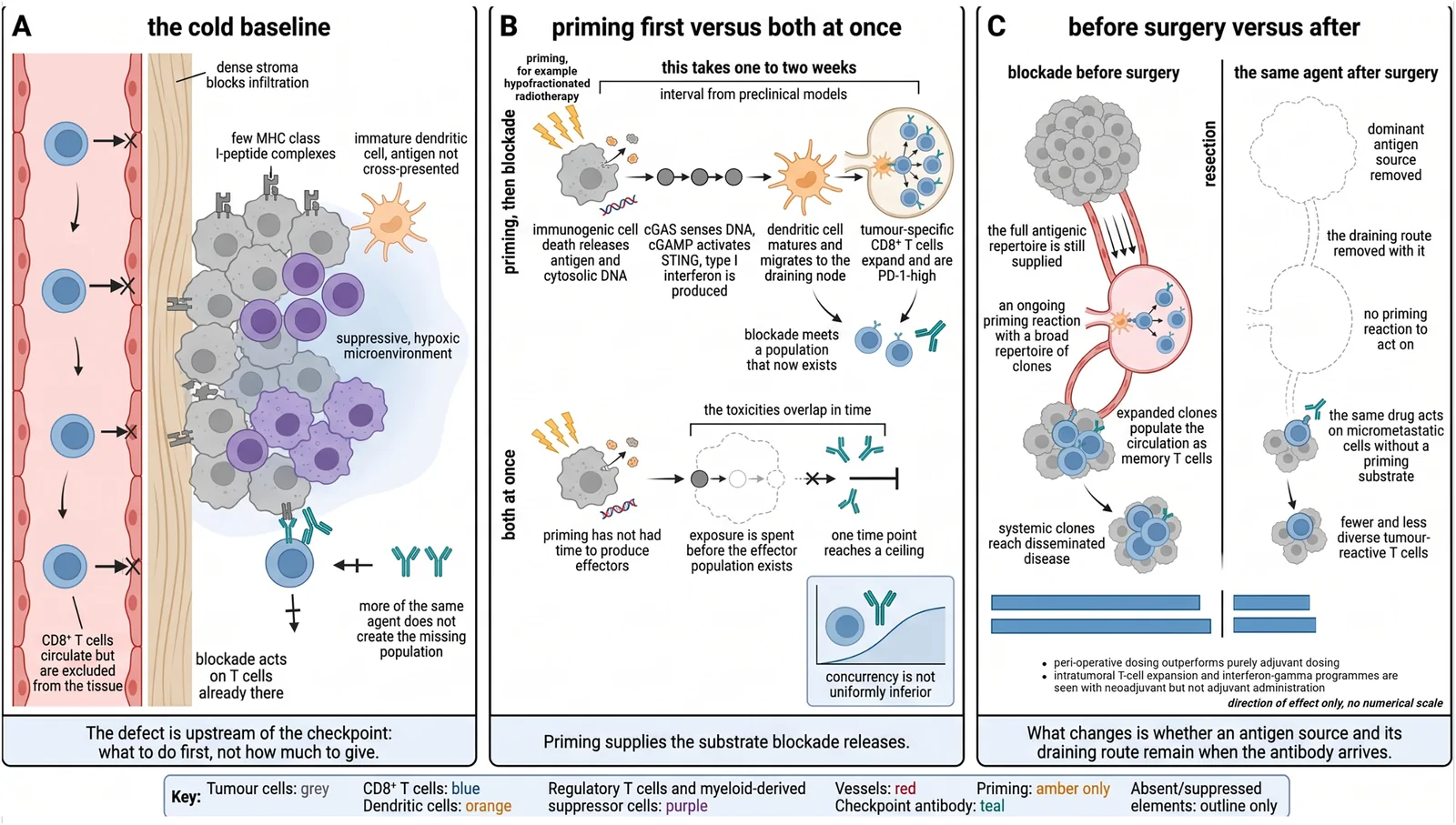
Why order matters at the cellular level: the cold baseline, priming before checkpoint blockade versus both agents at once, and blockade before versus after surgery. **(A)** The cold baseline shows the deficits that place the defect upstream of the checkpoint: few MHC class I-peptide complexes on tumor cells, an immature dendritic cell that does not cross-present antigen, a suppressive and hypoxic microenvironment containing regulatory T cells and myeloid-derived suppressor cells, and a dense stroma that blocks infiltration so that CD8 T cells circulate but are excluded from the tissue. Because checkpoint blockade acts on T cells that are already there, more of the same agent does not create the missing population. **(B)** The two schedules contrasted along a shared sequence. In the upper row, priming then blockade, hypofractionated radiotherapy causes immunogenic cell death and releases antigen and cytosolic DNA; cGAS senses that DNA and cGAMP activate STING, so type I interferon is produced; a dendritic cell matures and migrates to the draining node; tumor-specific CD8 T cells expand there and are PD-1-high; and blockade then meets a population that now exists. The bracket above the row marks that this takes 1 to 2 weeks, an interval taken from preclinical models. In the lower row, both at once, priming has not had time to produce effectors, so exposure is spent before the effector population exists, drawn as an outline because that population is absent, and a single time point reaches a ceiling while the toxicities of the two modalities overlap in time. The inset carries the counter-argument that concurrency is not uniformly inferior: where a modest infiltrate already exists, early blockade can pre-empt the counter-regulatory program rather than chase it, a scenario discussed in Section 11.1. **(C)** The same antibody is divided across a resection line. On the left, blockade before surgery, the full antigenic repertoire is still supplied, and blockade acts on an ongoing priming reaction with a broad repertoire of clones; the expanded clones populate the circulation as memory T cells and reach disseminated disease. On the right, the same agent after surgery, the dominant antigen source is removed, and its draining route is removed with it, leaving no priming reaction to act on, so the same drug acts on micrometastatic cells without a priming substrate and yields fewer and less diverse tumor-reactive T cells. What changes between the two sides is whether an antigen source and its draining route remain when the antibody arrives. The paired bars beneath the panel summarize the mechanistic reading of two clinical observations, namely, that peri-operative dosing outperforms purely adjuvant dosing and that intratumoral T-cell expansion and interferon-gamma programs are seen with neoadjuvant but not adjuvant administration (36–38); as labelled on the image, the bars indicate the direction of effect only and carry no numerical scale. The key beneath the panels applies throughout: tumor cells are gray, CD8 T cells are blue, dendritic cells are orange, regulatory T cells and myeloid-derived suppressor cells are purple, vessels are red, checkpoint antibody is teal, priming is amber, and absent or suppressed elements are drawn in outline only. The image is a mechanistic synthesis of the literature reviewed in Sections 3 to 5 and is not a depiction of a single experiment. Created in https://BioRender.com.

### 5.1 Priming kinetics: from antigen release to T-cell infiltration

Local priming, canonically by radiotherapy, initiates a defined kinetic cascade rather than an instantaneous change. Ionizing radiation induces immunogenic cell death, releasing tumor neoantigens and danger signals that convert a dying cell into an adjuvant for adaptive immunity (45, 46). This adjuvant signal is molecularly defined: dying tumor cells surface-expose calreticulin as an “eat-me” signal that promotes dendritic-cell engulfment, secrete ATP as a chemoattractant for antigen-presenting cells, and release the nuclear protein HMGB1, which engages Toll-like receptor 4 to license efficient antigen cross-presentation (46). It also upregulates MHC class I and broadens the presented peptide repertoire in a dose-dependent manner (47) and enhances dendritic-cell cross-presentation, shifting the microenvironment from immunosuppressive towards immunostimulatory. A central node in this cascade is innate sensing of radiation-induced cytosolic DNA: the cGAS–STING pathway in dendritic cells drives type I interferon production that is required both for spontaneous T-cell priming against tumors and for cross-priming of tumor-specific CD8^+^ T cells after irradiation (48). This sensing step imposes an upper bound on useful fraction size: above roughly 12–18 Gy, the exonuclease Trex1 is induced and degrades the cytosolic DNA that activates cGAS–STING, so repeated moderate fractions, rather than a single ablative dose, maximize interferon-β production and Batf3-dependent dendritic-cell priming (49). The fractionation schedule is, therefore, itself part of the timing logic: fractionated, but not single-dose, radiotherapy elicits the systemic (abscopal) immune-mediated effect when combined with checkpoint blockade (50), the mechanistic basis for the hypofractionated regimens (e.g., 8 Gy × 3) favored in immuno-radiotherapy (51). These events unfold over days to a few weeks and reconfigure the microenvironment through multiple, non-redundant mechanisms (52); priming also builds the structures that sustain a response, with low-dose radiotherapy plus anti-PD-1 increasing the number and maturity of intratumoral tertiary lymphoid structures (TLS) in preclinical models (53). The provenance of this interval should be stated precisely. In the murine models cited above, the newly primed effector wave becomes detectable within the tumor roughly 1 to 2 weeks after irradiation (48–50, 54), so the 1- to 2-week figure is derived from preclinical kinetics rather than from a human timing study. Human data are so far corroborative rather than confirmatory, and they are indirect. In patients with chemorefractory metastatic NSCLC receiving radiotherapy plus ipilimumab, radiation expanded CD8 T-cell clones recognizing a radiation-exposed neoantigen. An early rise in serum interferon-beta, together with early peripheral T-cell clonal dynamics, distinguished responders (objective response 18%, disease control 31%) (55). The pooled PEMBRO-RT and MDACC analysis likewise shows that checkpoint blockade begun within 1 week of the last fraction augments out-of-field response (56). Two caveats limit direct transfer to human tumors. The kinetics were measured in fast-growing transplantable murine tumors, whose immune turnover is compressed relative to human disease. The human corroboration rests on peripheral blood and radiographic surrogates rather than on serial intratumoral sampling of the effector wave itself. We, therefore, treat 1 to 2 weeks as a testable first approximation whose human optimum must be established empirically, most directly by randomizing the priming-to-checkpoint interval (Section 11), rather than as an established human window. The therapeutic implication is that checkpoint blockade is most likely to convert a response when it coincides with, rather than precedes, the wave of newly primed, tumor-reactive T cells.

### 5.2 The vascular-normalization window

A second clock controls whether primed T cells can physically enter the tumor. Anti-angiogenic therapy, delivered at vascular-normalizing rather than vessel-pruning doses, transiently remodels tumor vasculature to improve perfusion and lymphocyte infiltration—an effect first defined as a discrete “normalization window” (57). The decline of this curve, as depicted in **Figure 2**, is not an inference from adaptive resistance but a directly measured property of the window. In orthotopic brain tumors, VEGFR2 blockade improved perfusion and oxygenation only transiently. Radiation given during that interval was more effective than radiation given either before it or after it had closed, which defines the effect as a window rather than a plateau (42). Continued or higher-dose exposure beyond the window prunes rather than repairs vessels, so perfusion falls back and the immunological advantage is lost (43). Two mechanisms close this window, and both are distinct from the counter-regulation of Section 5.3. Sustained VEGF-pathway inhibition converts normalization into excessive regression, and the remodeled vasculature reverts once the normalizing stimulus is withdrawn. The underlying principle—that transiently repairing rather than starving tumor vasculature alleviates hypoxia, improves perfusion, and thereby licenses immune-effector entry—has been developed into a general rationale for combining anti-angiogenics with immunotherapy (58); beyond VEGF, angiopoietin-2 contributes to the abnormal, immunosuppressive vasculature, so co-targeting both pathways may widen the normalization window (58). The relationship is reciprocal: combined anti-VEGFR2 and anti-PD-L1 therapy induces intratumoral high endothelial venules that promote lymphocyte infiltration and activity in preclinical models, so vascular normalization and antitumor immunity reinforce one another when the two are engaged in the right relation (59). Low-dose anlotinib extends this principle, producing durable normalization that improves anti-PD-1 efficacy in preclinical models, whereas higher, still-tolerable doses are less effective—evidence that dose and timing, not merely drug exposure, define the immunologic benefit (41). Translating a “normalizing dose” to the clinic is not trivial because the window is narrow and patient-specific, but the principle (lower, perfusion-preserving doses rather than maximal vascular pruning) increasingly guides anti-angiogenic–immunotherapy design. Innate-immune agonism converges on the same mechanism: STING activation drives a biphasic vascular response and is maximally effective only when combined with VEGFR2 blockade or checkpoint inhibition (60), a behavior reproduced by STING-activating nanoparticles (61). Because these data are largely preclinical and clinical STING agonists have translated poorly to date, the vascular window is best regarded as a mechanistic rationale for sequencing rather than a validated clinical schedule—but the direction is consistent: entry must be licensed before or as effector T cells arrive.

### 5.3 Compensatory adaptive-resistance windows

Priming does not only open opportunities; it triggers counter-regulation on its own timescale. Antigen release and IFN signaling upregulate PD-L1 and mobilize regulatory T cells and myeloid-derived suppressor cells (62), creating a window of adaptive immune resistance that can blunt a delayed or mistimed intervention. This counter-regulation is measurable and time-resolved: fractionated radiotherapy upregulates tumor-cell PD-L1 dynamically over the first days after treatment, and PD-1/PD-L1 blockade delivered within that interval, rather than long afterwards, overcomes radiation-induced resistance in preclinical models, whereas delayed blockade does not (54). The counter-regulatory program is broad—encompassing not only PD-L1 induction but also the recruitment of regulatory T cells and myeloid-derived suppressor cells, upregulation of alternative inhibitory receptors such as TIM-3 and LAG-3 (63), and IDO-mediated tryptophan catabolism (64)—so a window opened by priming begins to close within days unless it is exploited. Metronomic, low-dose chemotherapy exploits the same window from the opposite direction, preferentially depleting regulatory T cells and myeloid-derived suppressor cells while sparing effector populations and exerting antiangiogenic and pro-immune effects at immunomodulatory rather than cytotoxic doses (65). Because these effects are dose- and schedule-dependent (low, frequent dosing favors the immunomodulatory over the cytotoxic phenotype), metronomic chemotherapy is itself a timing intervention rather than merely a gentler chemotherapy. The existence of this compensatory window is the strongest biological argument for scheduling checkpoint blockade to the moment the primed response peaks: too early and there is little to disinhibit; too late and adaptive resistance has reasserted itself.

### 5.4 The microbiome operates on a temporal, not instantaneous, scale

The systemic conditioning provided by the gut microbiome has its own, much slower clock. In responders to anti-PD-1 therapy, favorable microbial features accrue over time rather than immediately, and progressive enrichment of immunogenic taxa follows fecal microbiota transplantation (40, 66). The gut microbiome shapes antitumor immunity systemically—tuning dendritic-cell activation, the type I interferon tone, and the balance of circulating effector and regulatory T cells—so its influence is exerted on the host’s baseline immune set-point rather than on the tumor directly. This slow, cumulative dynamic implies that microbiome modulation is best initiated early and sustained as a background conditioning layer rather than deployed as a same-day adjuvant—a theme developed in Section 8.

These four clocks together describe a shared “immunologic window of opportunity.” Two clarifications are warranted. First, the word “shared” is not a claim that four modalities must be coordinated with one another. The argument is narrower and refers to a single decision: when to give the checkpoint inhibitor. Each clock independently constrains that one decision, and they constrain it in opposite directions. Priming sets a lower bound because before the effector wave arrives there is little for blockade to disinhibit. Adaptive counter-regulation sets an upper bound because a window left unexploited begins to close. Vascular normalization determines whether the disinhibited cells can physically enter, and microbiome conditioning determines whether a systemic response can be mounted at all. The interval that satisfies all four bounds simultaneously is what we mean by a shared window, and if no such interval existed the sequencing hypothesis would collapse into four separate scheduling problems. Second, the day 7 to 14 window shown in **Figure 2** is a synthesis of preclinical kinetics, not a measured human interval, and its components differ in strength. Its lower bound comes from the priming and adaptive-resistance data above. There PD-L1 rises on tumor cells within days of fractionated radiotherapy, and blockade delivered inside that interval, but not later, overcomes radiation-induced resistance (54). Its upper bound is set by adaptive resistance: beyond roughly day 14, delayed blockade no longer shows benefit. The microbiome curve is deliberately not bounded by the same band since it is a slow background layer accruing over weeks (40, 66). The schema, therefore, starts conditioning one to several weeks earlier and sustains it, rather than timing it to days 7 to 14. The band is labelled as a preclinically derived hypothesis to be tested. The central proposition of this review is that effective cold-tumor therapy aligns each intervention to this window: prime to open it, inhibit checkpoints while it is open, and condition the host systemically so that the window can form at all. The following three sections examine each mechanistic axis, in turn, with explicit attention to what is known and not known about its optimal timing.

## 6 Mechanisms of microenvironment priming and their kinetics

Priming is the step that opens the immunologic window: it converts an antigen-poor, poorly perfused, suppressed lesion into one that a checkpoint inhibitor can exploit. Radiotherapy is the most clinically mature primer, but anti-angiogenic, innate-immune, oncolytic, and metronomic-chemotherapy approaches act on the same window and are reviewed here with explicit attention to whether the clinical evidence actually tests timing or only combination (**Table 2**).

**Table 2:** Cross-mechanism evidence on the timing of interventions in cold tumors.

Mechanistic axis	Representative evidence	Design	Key outcome	Tests timing?	Caveat	Ref.
Priming (RT)	RT + ICI network meta-analysis, 24 RCTs/9,480 patients	Sequential vs. concurrent	Sequential OS HR 0.81, PFS HR 0.73; concurrent did not reach the same benefit; grade ≥3 OR 1.02	Yes (seq vs. conc)	Sequencing contested; confirmatory RCTs ongoing	(16)
Priming (RT)	PEMBRO-RT	Sequential (SBRT→pembrolizumab <1 week)	PD-L1-negative subgroup OS HR 0.48 (95% CI 0.24–0.99)	Yes (sequential arm)	Exploratory subgroup; trial missed primary endpoint	(35)
Priming (RT)	PACIFIC/COAST consolidation	Sequential consolidation	Consolidation ICI after chemoradiotherapy improves survival	Partly (consolidation structure)	Not a head-to-head timing comparison	(17, 18)
Priming (anti-angiogenic/innate)	Low-dose anlotinib; STING agonism	Preclinical	Vascular-normalization window; enhances anti-PD-1	Indirect (mechanism only)	Mostly murine; STING agonists translate poorly	(41, 60)
Priming (anti-angiogenic, clinical)	APOLLO (anlotinib + penpulimab, HCC)	Concurrent (day 1)	OS 16.5 vs. 13.2 mo (HR 0.69); PFS 6.9 vs. 2.8 mo (HR 0.52)	No (concurrent)	Concurrent design—not timing evidence	(78)
Priming (vaccine)	Autogene cevumeran (PDAC); KEYNOTE-942 (melanoma)	Sequential (post-resection anti-PD-L1→vaccine); adjuvant RCT	Neoantigen-specific T cells tracking delayed recurrence; melanoma RFS HR 0.56	Partly (adjuvant/sequential positioning)	Vaccine timing not yet head-to-head; melanoma not cold	(74, 75)
Checkpoint	Cadonilimab, COMPASSION-03	Single-arm	PD-1/CTLA-4 bispecific: manageable safety + antitumor activity	No (combination)	Single-arm, early phase	(83)
Microbiome	Responder FMT + anti-PD-1	Concurrent FMT+ICI	Overcame resistance (clinical benefit 6/15; objective responses 3/10)	No (concurrent)	Small phase 1	(94, 95)
Microbiome	First-line FMT/probiotic: MIMic, PERFORM, CBM588	First-line/randomized	Feasible; positive signals (CBM588 PFS 12.7 vs. 2.5 mo, HR 0.15)	No (concurrent)	Single-arm/small-n	(40, 96, 97)
Microbiome (negative control)	SER-401/MCGRAW	Antibiotic preconditioning before ICI	Disrupted microbiome, impaired systemic immunity	Yes (negative): mistiming harms	Wrong sequence → harm	(19)

Representative clinical and preclinical evidence bearing on the timing of each priming or conditioning modality relative to checkpoint blockade in cold tumors. The “Tests timing?” column states whether each study actually varied sequence or interval (Yes), tested only the combination without varying timing (No), gave indirect or mechanism-only support, or served as a negative (mistiming) control; it shows that, to date, only radiotherapy–ICI scheduling and the antibiotic-preconditioning control directly interrogate timing, whereas most other entries establish combination efficacy alone. All quantitative outcomes were verified against the primary publications.

DCR, disease control rate; FMT, fecal microbiota transplantation; HCC, hepatocellular carcinoma; HR, hazard ratio; ICI, immune checkpoint inhibitor; mo, months; mOS/mPFS, median overall/progression-free survival; OR, odds ratio; ORR, objective response rate; OS, overall survival; PDAC, pancreatic ductal adenocarcinoma; PFS, progression-free survival; RCT, randomized controlled trial; RFS, recurrence-free survival; RT, radiotherapy; SBRT, stereotactic body radiotherapy; STING, stimulator of interferon genes.

### 6.1 Radiotherapy as the anchor of priming

The trial that best isolates the sequencing logic of radiotherapy is PEMBRO-RT. In this randomized phase 2 study, stereotactic body radiotherapy (SBRT; 24 Gy in three fractions, i.e., 8 Gy × 3) was delivered sequentially, with pembrolizumab beginning within 1 week of the last fraction; although the trial did not meet its prespecified primary endpoint, the benefit was concentrated in the PD-L1-negative (cold) subgroup, with an overall-survival hazard ratio of 0.48 (95% CI 0.24–0.99) (35). The clinical reality of radiotherapy-induced systemic immunity was first documented as the abscopal effect, regression of non-irradiated metastases, which, in a melanoma patient receiving ipilimumab plus radiotherapy, tracked with antigen-specific antibody and peripheral T-cell responses (67) and was subsequently reproduced prospectively when local radiotherapy was combined with granulocyte–macrophage colony-stimulating factor (abscopal responses in roughly a quarter of patients with metastatic solid tumors) (68). A pooled analysis of PEMBRO-RT and the concurrently dosed MDACC trial (*n* = 148) found that adding radiotherapy roughly doubled the out-of-field response rate (42% vs. 20%), providing landmark evidence that local priming augments systemic checkpoint response (56). That the out-of-field effect requires an intact immune system and is amplified by checkpoint blockade confirms its immunological, rather than merely cytoreductive, basis. Multisite stereotactic radiotherapy with pembrolizumab is similarly feasible and active across solid tumors (69). Small sequential case series point the same way—in unresectable HCC, SBRT followed by anti-PD-1 produced high tumor-response rates in an early proof-of-concept report (70). The recurring theme is that radiotherapy’s value in cold disease is less as a local cytoreductive add-on than as a systemic primer whose effect is realized through subsequent checkpoint blockade. Two practical corollaries follow for how radiotherapy should be delivered when the goal is priming rather than local control: hypofractionated, immunogenic dosing is preferred over protracted low-dose-per-fraction schedules (Section 5.1), and confining the target to a limited volume helps spare the circulating and nodal lymphocytes whose depletion by large-field, protracted radiotherapy can itself blunt the systemic antitumor response.

### 6.2 Sequential versus concurrent: the clinical signal and its caveats

The strongest quantitative support for sequencing over simultaneous addition comes from meta-analysis. The network meta-analysis of 24 randomized trials (9,480 patients) found that sequential ICI administered after radiotherapy improved overall survival (HR 0.81, 95% CI 0.72–0.92) and progression-free survival (HR 0.73, 95% CI 0.64–0.84) versus control and was superior to concurrent administration for progression-free survival (HR 0.81, 95% CI 0.67–0.97), whereas concurrent radiotherapy plus ICI showed no significant benefit over control (16). The PACIFIC-derived consolidation paradigm operationalizes this at a practice-changing scale: durvalumab is given as consolidation after completion of chemoradiotherapy, structurally a prime-then-inhibit sequence rather than concurrent co-administration, and delivered a durable overall-survival benefit in unresectable stage III NSCLC (17), a design extended in the COAST platform (18). This evidence should be read with appropriate caution: the sequencing question remains actively contested, the strongest evidence is a network meta-analysis rather than a head-to-head sequencing trial, and confirmatory randomized trials are ongoing, so the directional signal (sequential ≥ concurrent in cold disease) is best read as a date-stamped hypothesis, not a settled rule. The most recent randomized evidence in this axis favors the consolidation principle over concurrency. In the final analysis of the COAST platform study, durvalumab-based consolidation, begun after chemoradiotherapy, either alone or with oleclumab or monalizumab, sustained its advantage over durvalumab alone (objective response 35.0% and 40.3% versus 23.9%; progression-free survival hazard ratio 0.59 and 0.63; overall survival hazard ratio 0.69 and 0.77) (71). Mechanistically, there are two reasons concurrent dosing might underperform: the checkpoint inhibitor is partly spent on a microenvironment that has not yet been primed, and newly activated T cells in the circulation and draining nodes are exposed to radiation-induced lymphodepletion. Both considerations favor delivering blockade into, rather than across, the priming interval, and several randomized trials now testing explicit sequencing schedules should convert this inference into direct evidence within the next few years.

Beyond efficacy, sequencing carries a clinician-facing rationale that concurrent stacking cannot claim: separating modalities in time also separates their toxicities. Concurrent radio-immunotherapy superimposes radiation pneumonitis on immune-related pneumonitis, whereas a prime-then-inhibit schedule spaces these liabilities and may improve deliverability in frail patients. Reassuringly, no increase in treatment-related toxicity was observed when radiotherapy preceded pembrolizumab in PEMBRO-RT (35), and grade ≥3 adverse events were a prespecified endpoint of the network meta-analysis, which showed no increase in toxicity with sequential versus concurrent dosing (odds ratio 1.02) (16). Here the mechanistic and the tolerability arguments point in the same direction. For the frail, heavily pre-treated patients who typify cold disease, a schedule that is at once more effective and more tolerable is not a marginal refinement but often the difference between a deliverable regimen and none.

### 6.3 Broadening the primer: anti-angiogenic, innate, oncolytic, vaccine, and metronomic approaches

Priming is not limited to radiotherapy. Vascular-normalizing anti-angiogenic therapy can prepare the same microenvironmental window (Section 5.2): low-dose anlotinib produces durable normalization that enhances anti-PD-1 activity preclinically (41), and adding an anti-angiogenic to checkpoint blockade improves outcomes even in PD-L1-low, poorly inflamed NSCLC in the IMpower150 trial (progression-free survival 8.3 vs. 6.8 months, hazard ratio 0.62; overall survival 19.2 vs. 14.7 months), with benefit retained in PD-L1-low and liver-metastatic subgroups (72)—clinical evidence that the vascular axis matters in cold disease. Innate-immune agonism converges on the same mechanism, with STING activation reproducing vascular normalization and TLS induction in mouse models (60, 61). Oncolytic virotherapy offers a further clinically validated priming route: intratumoral talimogene laherparepvec increases CD8^+^ T-cell infiltration and interferon-γ signaling and, when followed by pembrolizumab, produced responses that were not predicted by baseline CD8 density—the hallmark of a primer that manufactures, rather than merely exploits, a pre-existing infiltrate (73). Cancer vaccines are a further antigen-supplying primer whose sequencing logic is explicit in that personalized neoantigen vaccines manufacture a tumor-specific T-cell repertoire *de novo*. In resected high-risk melanoma, adjuvant mRNA-4157 (V940) plus pembrolizumab prolonged recurrence-free survival over pembrolizumab alone (hazard ratio 0.56) in the randomized KEYNOTE-942 trial (74), and, most relevant to cold disease, an individualized mRNA vaccine (autogene cevumeran) delivered sequentially after resection and anti-PD-L1 in pancreatic ductal adenocarcinoma induced durable neoantigen-specific T cells that tracked with delayed recurrence (75)—proof that priming can raise an antigen-specific response in one of the coldest human tumors and that the vaccine’s position relative to checkpoint blockade is a deliberate design variable rather than an afterthought. Metronomic, low-dose chemotherapy is a further especially accessible primer, lowering the regulatory T-cell and myeloid-derived-suppressor-cell set-point while contributing its own immunogenic-cell-death and antiangiogenic effects (46, 65, 76). That such priming can matter clinically in genuinely cold disease is suggested by REGONIVO, in which regorafenib plus nivolumab achieved a 36% response rate (median progression-free survival 7.9 months) in microsatellite-stable colorectal cancer, a tumor type essentially refractory to checkpoint monotherapy, albeit with concurrent rather than sequenced dosing (77). Indeed these modalities are, as a class, mechanistically attractive but clinically immature for the sequencing claim: pivotal anti-angiogenic-plus-ICI trials such as APOLLO (anlotinib plus penpulimab in HCC) used concurrent day-1 co-dosing and, therefore, established the efficacy of the combination without testing prime-then-inhibit timing (78). How a primer reaches the tumor also shapes the interval it creates since formulation and delivery route govern where an agent is present and for how long (79), and computational design is now being applied to chemo-immunotherapy scheduling and to nanocarrier engineering, where the dose-and-interval permutations exceed what empirical testing can enumerate (80, 81). Positioning anti-angiogenics, innate agonists, and metronomic chemotherapy as primers, rather than as simultaneous partners, remains a rationale-driven hypothesis that a timing-based framework makes explicit and testable.

## 7 Deploying checkpoint blockade within the window

Checkpoint inhibition is the destination of the sequence, not its starting point. In genuinely cold tumors, ICI monotherapy rarely converts a response—as the HCC failures of CheckMate 459 and KEYNOTE-240 illustrate (Section 1) (5, 6)—because the yield of checkpoint blockade is set by the microenvironmental state created before it: the newly primed, tumor-reactive T cells and the transiently normalized vasculature that lets them enter (Sections 5.1–5.2). Framed temporally, checkpoint blockade is a relay baton that must be handed off while the primed window is open: given before priming, there is little pre-existing immunity to disinhibit; given after adaptive resistance has reasserted itself (Section 5.3), its substrate has already contracted. This reframing explains an otherwise puzzling clinical observation—that intensifying checkpoint blockade in cold tumors, whether by higher doses or additional agents, so often fails to help: without a primed substrate, there is simply little for a more powerful inhibitor to release.

Two considerations refine how checkpoint blockade is deployed within this window. First, the depth of inhibition can be matched to the breadth of the primed repertoire. Dual checkpoint blockade delivers durable long-term survival in advanced melanoma—a 5-year overall survival of 52% with nivolumab plus ipilimumab versus 26% with ipilimumab alone (13)—and, in cold disease, a single priming dose of tremelimumab added to durvalumab (the STRIDE regimen) improved survival over sorafenib in unresectable HCC (overall-survival hazard ratio 0.78; 36-month survival 30.7% vs. 19.8%) with a differentiated safety profile (82). STRIDE is itself instructive for the timing argument: it delivers only one dose of the anti-CTLA-4 agent as an immunological “prime” rather than continuously, embodying the logic of applying the deepest blockade briefly and early. PD-1 × CTLA-4 bispecific antibodies extend this logic: cadonilimab, a tetravalent bispecific, showed manageable safety and antitumor activity across advanced solid tumors in COMPASSION-03 (83), with a trans-binding design that concentrates activity where both targets are co-expressed (84). By focusing dual blockade at sites of PD-1/CTLA-4 co-expression, such designs may permit deeper inhibition with less systemic toxicity than two separate antibodies—an attractive property when the deepest blockade is wanted only briefly and only within the primed window. The corollary is a decision rule rather than a default: dual or bispecific blockade is warranted when priming is expected to generate a broad new repertoire (after high-dose or multi-site irradiation, for instance), whereas a narrowly primed tumor may gain only toxicity from deeper blockade, so the depth of inhibition should track the breadth of priming. The division of labor is well defined mechanistically: radiation, CTLA-4 blockade, and PD-1/PD-L1 blockade act through non-redundant nodes—radiation diversifies the intratumoral T-cell-receptor repertoire, and anti-PD-1/PD-L1 reverses exhaustion of the resulting cells (85). The CTLA-4 axis is distinctive: beyond releasing an inhibitory signal on effector T cells (86), much of the antitumor activity of anti-CTLA-4 in preclinical models derives from Fcγ-receptor-dependent depletion of intratumoral regulatory T cells (87), and single-cell profiling confirms that the two checkpoints act on distinct cellular substrates—anti-PD-1 chiefly expands exhausted-like CD8^+^ subsets, whereas anti-CTLA-4 additionally mobilizes an ICOS^+^ Th1-like CD4^+^ effector population (88). Because CTLA-4 blockade both expands and prunes the effector-versus-regulatory balance, it is most relevant precisely when priming has just generated a new neoantigen-reactive repertoire—again arguing for placement of the deepest blockade after, not before, the priming step. Second, the sequencing hypothesis for checkpoint blockade itself remains to be tested directly: pivotal combination trials such as APOLLO used concurrent day-1 co-dosing (Section 6.3) and, therefore, demonstrated that the combination works without isolating the contribution of timing (78). A timing-based framework makes this an explicit, falsifiable claim—that scheduling checkpoint blockade to the post-priming window will outperform simultaneous administration in cold disease—rather than an implicit assumption.

## 8 Systemic conditioning: the microbiome as a slow background layer

The third axis acts not on the local tumor but on the host, setting whether an antitumor response can be mounted at all. It is best conceived not as a co-equal, tightly scheduled partner to radiotherapy and checkpoint blockade but as a slow background conditioning layer that must be established early and sustained. Its evidence base progresses cleanly from correlation to causation to intervention. Responders to anti-PD-1 therapy show higher gut microbial diversity and enrichment of specific taxa (66, 89). The relationship is causal rather than merely associative: transfer of responder microbiota into germ-free mice enhances systemic and antitumor immunity, and defined commensals reconstitute checkpoint sensitivity—*Bifidobacterium* restores anti-PD-L1 efficacy, specific Bacteroides are required for anti-CTLA-4 activity, and an 11-strain consortium induces IFN-γ^+^ CD8 T cells that augment ICI in tumor models (90–92). Microbe-derived metabolites supply a molecular mechanism, with *Bifidobacterium pseudolongum* enhancing checkpoint response through the metabolite inosine acting on T-cell adenosine A2A receptors (93). Interventionally, responder-derived fecal microbiota transplantation (FMT) combined with anti-PD-1 overcame resistance in a subset of refractory melanoma patients in two independent trials (clinical benefit in six of 15 and objective responses in three of 10 patients, respectively) accompanied by increased intratumoral CD8^+^ activation and a reduced frequency of interleukin-8-expressing immunosuppressive myeloid cells (94, 95). The axis now extends into the first-line setting: FMT plus first-line anti-PD-1 was feasible with encouraging activity in treatment-naive melanoma (40), encapsulated donor FMT met its safety endpoint in treatment-naive renal cell carcinoma (PERFORM) (96), and the live bacterial product CBM588 improved outcomes when added to nivolumab–ipilimumab in a randomized trial (97). Standardizing the microbial product (donor selection, defined multi-strain consortia, or engineered strains) remains a central translational hurdle since the “dose” in this axis is a living community rather than a defined molecule. Rationally programmed approaches illustrate how far this axis may eventually be steered: engineered commensals can be programmed to release tumor antigens or immunostimulatory payloads within the tumor or gut, converting the microbiome from a permissive background into an active, targetable delivery vehicle whose activity can be switched in time rather than left constitutive (98, 99), while dietary fiber, by feeding short-chain-fatty-acid-producing taxa, offers a low-cost and sustainable means of shifting the same background in a favorable direction (100).

What distinguishes this axis from a simple co-medication is its clock. The microbiome–immune relationship strengthens over time rather than instantaneously—favorable features and immunogenic taxa accrue across weeks in responders (40, 66)—so conditioning is best initiated early and sustained beneath the local priming–inhibition sequence rather than added on the day of checkpoint blockade. The clearest demonstration that timing matters here is a negative one: in a randomized melanoma trial, aggressive antibiotic (vancomycin) pre-conditioning immediately before ICI disrupted the microbiome and impaired systemic immunity with incomplete recovery at the time of checkpoint initiation (19). Diet points the same way, with higher fiber intake, but not over-the-counter probiotic use, associated with improved ICI outcomes (100). The broader lesson is that this axis punishes disruption more than it rewards acute manipulation—protecting an existing favorable microbiome may matter as much as attempting to install a new one. For the microbiome, as for radiotherapy and checkpoints, the wrong sequence subtracts; but unlike those local steps, its contribution is a sustained baseline rather than a discretely timed pulse.

### 8.1 Testing the microbiome-first hypothesis: what the existing trials do not do and what a lead-in trial would require

The candor this axis requires is that the timing claim made for it is the least tested in this review. Across the prospective microbiome-plus-checkpoint literature, essentially every trial delivers the microbial intervention concurrently with checkpoint blockade or immediately before the first dose, and none randomizes lead-in against co-administration. The single-arm responder–donor FMT studies in melanoma gave transplant with or days before the first checkpoint dose and then continued it on treatment (94, 95). The randomized first-line melanoma trial of healthy-donor FMT likewise began transplant shortly before checkpoint initiation and maintained it throughout (40). The renal-cell probiotic study added CBM588 alongside nivolumab and ipilimumab from cycle 1 (97). The multi-cohort FMT experience in checkpoint-refractory disease again used concurrent delivery (96). Counting generously, the number of prospective trials in which the microbiome axis is deliberately conditioned for a defined interval before checkpoint blockade, with the interval itself as the variable under study, is zero. What the field has instead is one informative negative experiment about timing. Randomized vancomycin pre-conditioning immediately before checkpoint blockade disrupted the microbiome and left systemic immunity incompletely recovered when the checkpoint inhibitor was given (19). That result establishes that the interval before checkpoint blockade is not immunologically inert, which is precisely why it deserves a trial rather than an assumption.

A trial able to test the microbiome-first hypothesis has a small number of non-negotiable design features. Its randomized comparison must be of schedule and not of agent. Both arms receive an identical microbial intervention at identical dose and duration. The only difference is whether conditioning starts a defined interval before the first checkpoint dose or on the same day. The interval should be pre-specified from the kinetics rather than chosen for convenience. Favorable features accrue over weeks in responders (40, 66), which argues for 2 to 4 weeks as the first interval to test, with a second, longer arm if a platform design permits. Because the intervention is a living consortium rather than a fixed molecule, engraftment must be treated as the mechanistic gate. That means serial stool metagenomics at baseline, at the end of the lead-in, on the day of the first checkpoint dose, and at the first response assessment. Donor-strain engraftment and diversity change are reported as co-primary mechanistic endpoints alongside the clinical endpoint. A trial that reports only clinical outcome cannot distinguish a failed hypothesis from a failed engraftment. Sample size should be powered for the schedule contrast, not for the agent effect, which is the more demanding requirement and the reason such a trial is best embedded in a platform with a shared control arm (Section 11).

Four confounders make any microbiome timing readout harder to interpret than a radiotherapy interval, and each has to be handled in the protocol rather than in the discussion. Diet is a continuous, unblinded exposure acting on the same axis as the intervention. Higher fiber intake, but not over-the-counter probiotic use, tracks with checkpoint benefit (100), so dietary intake needs prospective quantification and, ideally, stratification. Antibiotic history is the strongest known modifier and is not a simple yes-or-no covariate because recency, spectrum, and cumulative exposure differ in effect. A defined pre-randomization window, with stratification on exposure inside it, is the minimum. Baseline gut diversity and community composition determine how much headroom an intervention has since a favorable starting community may leave little to improve. Both should be stratification factors rather than *post-hoc* subgroups. Concurrent cytotoxic chemotherapy perturbs the microbiome directly and in cold tumors is often unavoidable. That argues for restricting the first lead-in trial to a chemotherapy-free backbone or at minimum for holding the chemotherapy schedule identical across arms. To these should be added center effects on donor material and product handling, which are best controlled by a single characterized donor pool. Until a trial with these features reports, the microbiome-first element of any sequencing schema should be presented as the most speculative of its three axes, and we present it as such.

## 9 Reading the window: dynamic biomarkers

A therapy built on timing is only as good as its ability to tell when the window is open. The biomarkers used to select patients today are almost all static (measured once, before treatment), whereas the window that Sections 5–8 describe opens and closes over days to weeks. Bridging that gap is the central biomarker challenge for any sequencing strategy.

Static baseline markers remain valuable for setting the stage. Tumor mutational burden is associated with ICI benefit across histologies, albeit with tumor-type-specific thresholds (30); a T-cell-inflamed, IFN-γ-related gene-expression profile predicts response to PD-1 blockade (101); the density of CD8^+^ T cells at the invasive margin is itself among the strongest single baseline correlates of response (22); and TMB and the inflammation signature capture largely independent axes, so their joint assessment improves prediction (102). The presence and maturity of tertiary lymphoid structures, and the B-cell programs within them, provide a functional histological readout of a productively “warming” microenvironment and correlate with checkpoint response across melanoma, sarcoma, and other histologies (103–105). Yet all of these require baseline tumor tissue that is often scant or inaccessible in cold lesions, and none captures the state of the microenvironment after an intervention has begun. These measures answer “is this tumor likely to be ignitable?”—a necessary first question, but not the one a sequencing decision turns on.

The decision that timing forces is different: has the window actually opened yet, and is it still open? Answering it requires serial, dynamic readouts. On-therapy transcriptomics show that it is the induced IFN-γ signaling program, not merely its baseline level, that tracks with clinical response, implying that the relevant signal is a change over time rather than a snapshot (106). At single-cell resolution, the CD8^+^ T-cell states that separate responders from non-responders can be resolved directly, with a TCF7^+^ progenitor-exhausted population predicting benefit (107). Minimally invasive dynamic markers are especially attractive because they can be sampled repeatedly: early kinetics of peripheral blood immune subsets and of circulating tumor DNA (ctDNA) reflect the reshaping of the tumor microenvironment during checkpoint blockade and outperform static TMB and PD-L1 in predicting outcome (108). Functional imaging adds a further non-invasive axis: early changes in tumor perfusion or metabolic activity after priming could, in principle, report that the vascular window has opened before checkpoint blockade is committed. In a sequencing framework, such readouts have concrete roles—ctDNA kinetics to time the post-priming trough of tumor burden, on-treatment IFN-γ or TLS-maturation signatures to confirm that a window has opened before checkpoint blockade is committed, and longitudinal stool profiling to verify that the microbiome layer is trending favorably rather than being eroded. The intensity of such a serial tissue-plus-liquid sampling schedule is itself a feasibility barrier to be tested rather than assumed. No such dynamic, decision-linked biomarker strategy has yet been prospectively validated for sequencing; building and testing one is itself a priority and the natural companion to the trial designs discussed in Section 11. Because these candidates differ widely in how close each is to informing an actual decision, **Table 3** grades them by readiness rather than listing them side by side. Only two are measurable now with enough standardization to be pre-specified in a prospective trial: circulating tumor DNA kinetics and on-treatment CD8 density at the invasive margin. Even these lack agreed thresholds. The rest are either mechanistically informative but not decision-grade or hypothesis-generating. Stating the tier explicitly matters for trial design since an adaptive schedule trial that drops arms on a tier 3 readout would be adapting on noise.

**Table 3:** A tiered appraisal of candidate dynamic biomarkers for timing the priming-to-blockade interval.

Tier	Readout	What it is proposed to report	Why it is not yet actionable	What would move it up a tier	Ref.
Tier 1: ready for prospective use as a stratification or exploratory endpoint	Circulating tumor DNA kinetics	Early molecular response or clearance after the primer, as a lead indicator that the window has opened	Assay platforms, sampling intervals, and response thresholds are not harmonized, and shedding differs by histology and disease burden	Pre-locked platform, sampling schedule, and threshold, tested against a clinical endpoint in a single trial	(101–103)
Tier 1: ready for prospective use as a stratification or exploratory endpoint	On-treatment biopsy: CD8 density at the invasive margin	Direct confirmation that the primer converted the margin	Invasive, single-site, and confounded by sampling variability in heterogeneous lesions	Standardized scoring with a pre-stated cutoff, plus paired-lesion sampling to quantify heterogeneity	(35, 36)
Tier 2: mechanistically informative, not yet decision-grade	Peripheral T-cell clonal dynamics	Expansion of primer-induced clones as evidence that priming produced a usable repertoire	Reported associations come from small correlative sets, and no threshold distinguishes responders prospectively	Replication in an independent prospective cohort with a pre-specified expansion metric	(55, 105)
Tier 2: mechanistically informative, not yet decision-grade	Type I interferon signature and early serum interferon-beta	That the primer engaged the cytosolic sensing pathway on which the interval depends	Kinetics are rapid, sampling windows are narrow, and induction does not always translate to clinical benefit	Dense early sampling in a schedule-randomized trial, tying the signature to the interval rather than to outcome alone	(48, 50, 55)
Tier 2: mechanistically informative, not yet decision-grade	Imaging-derived perfusion as a surrogate for the normalization window	Whether the vascular window is open and, therefore, whether entry is possible	Perfusion imaging is not standardized across platforms, and the human window has not been mapped	Prospective serial imaging with a pre-defined open-window definition, anchored to a randomized interval contrast	(41–43)
Tier 3: hypothesis-generating only	Stool metagenomics: diversity and donor-strain engraftment	Whether systemic conditioning has taken hold and when	Neither an engraftment threshold nor a required duration is defined, and diet, antibiotics, and chemotherapy all perturb the readout	The lead-in trial of Section 8.1, with engraftment as a pre-specified mechanistic co-primary endpoint	(40, 66, 100)
Tier 3: hypothesis-generating only	Inhibitory-receptor and immunosuppressive-program induction	That the counter-regulatory clock has started, setting the upper bound of the window	Demonstrated largely preclinically; no validated human assay reports window closure in real time	A human time-course study mapping induction against a randomized priming-to-blockade interval	(62–64)

Candidate readouts are graded by how close each is to informing a scheduling decision, rather than by how often it is discussed. Tier 1 readouts are measurable now and could be pre-specified in a prospective trial as stratification factors or exploratory endpoints. Tier 2 readouts carry mechanistic weight but lack the standardization or the prospective thresholds that a decision rule requires. Tier 3 readouts remain hypothesis-generating. No readout in any tier is currently qualified to govern the timing of checkpoint blockade in routine care. The adaptive design of Section 11.2 must, therefore, pre-lock whichever readout it uses and report its operating characteristics rather than assume its validity.

CD8, cluster of differentiation 8; ctDNA, circulating tumor DNA.

## 10 Toward integrated, time-sequenced strategies

### 10.1 A field converging on integration

The preceding sections make a general case that ordering governs outcome in cold disease. The field is now beginning to act on it, and several integrated, time-sequenced strategies are being explored in parallel—each unifying two or more of the mechanistic axes reviewed above along a deliberate time axis. Consolidation immunotherapy after chemoradiotherapy is the most mature, embedding a prime-then-inhibit sequence in standard practice at the population scale (17, 18). Neoadjuvant-first paradigms, in which checkpoint blockade is delivered before definitive surgery to exploit an intact antigen source, have moved from concept to randomized evidence across NSCLC, melanoma, and glioblastoma (36–39). Microbiome-lead-in strategies, in which conditioning precedes or accompanies first-line checkpoint blockade, are being tested prospectively (40, 96). Newer effector classes are moving in the same temporal direction: in small-cell lung cancer, among the coldest tumors, the DLL3 bispecific T-cell engager tarlatamab is being delivered as maintenance after first-line chemo-immunotherapy rather than concurrently (109), and individualized neoantigen vaccines are positioned adjuvantly, after resection and relative to checkpoint blockade (Section 6.3) (75). At the conceptual level, the temporal dynamics of immunotherapy across modalities have been mapped as a general organizing principle (44). Other efforts modify the microenvironment before immunotherapy by different means: total neoadjuvant regimens that stack chemotherapy, stromal-targeting agents, and radiotherapy ahead of definitive treatment in pancreatic cancer, for example, apply the same prime-then-act logic to a heavily excluded tumor. What unites these otherwise disparate strands is a deliberately staged sequence rather than a simultaneous stack. What none of these yet provides is a single framework that unifies microenvironment priming, checkpoint deployment, and microbiome conditioning on one time axis, specific to cold tumors, and stated concretely enough to be falsified.

### 10.2 One such exploration: a three-pillar, time-sequenced schema

One attempt to fill that gap, among what will need to be several, is a three-axis arrangement we term TMEp-CI-M, which orders the mechanisms of Sections 6–8 along the immunologic window of Section 5 (**Figure 4**). Tumor microenvironment priming (TMEp)—radiotherapy at its core, optionally reinforced by vascular-normalizing anti-angiogenics or metronomic chemotherapy—opens the window; checkpoint inhibition (CI), matched in depth to the primed repertoire, is delivered into it; and microbiome modulation (M) runs continuously beneath both as the background conditioning layer of Section 8. The organizing claim is not that these agents should be combined, that is the addition paradigm, but that their order and interval determine whether a cold tumor is ignited. The three axes are chosen because they address non-overlapping deficits of the cold state—priming supplies antigen and access, checkpoint blockade removes the brake on the resulting T cells, and microbiome conditioning sets the systemic permissiveness for a response—so that, unlike partners that duplicate a single mechanism, each contributes something the others cannot.

**Figure 4:**
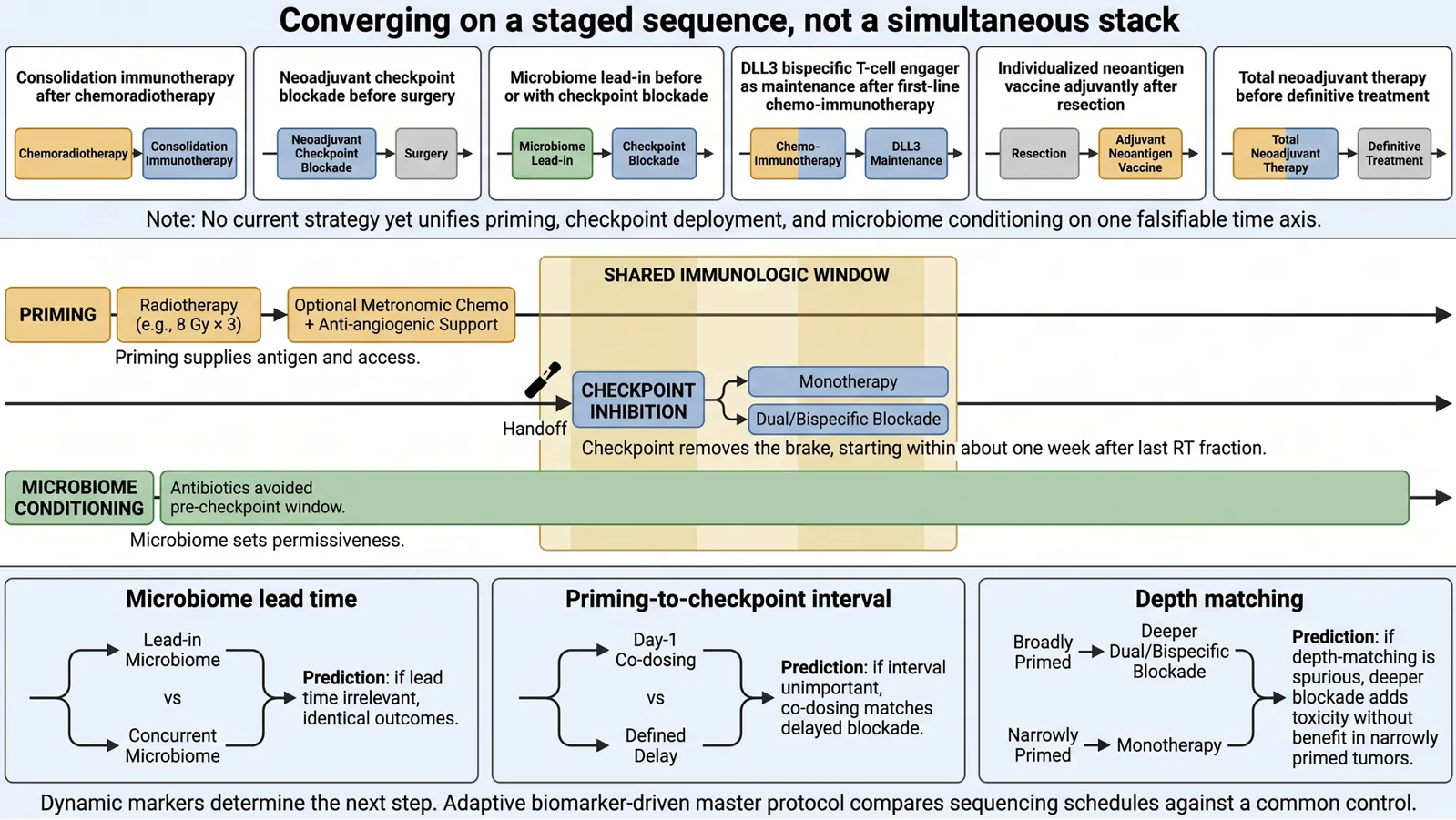
Toward integrated, time-sequenced strategies for cold tumors, and one concrete, falsifiable three-axis schema. The upper band presents hypothesis-generating examples in which the field is converging on ordered rather than simultaneous therapy, each shown as a staged token on a shared time arrow: consolidation immunotherapy after chemoradiotherapy (17, 18), neoadjuvant-first checkpoint blockade (36–38), microbiome lead-in before or with first-line checkpoint blockade (40), the DLL3 bispecific T-cell engager tarlatamab given as maintenance after first-line chemo-immunotherapy rather than concurrently (109), individualized neoantigen vaccines positioned adjuvantly after resection relative to checkpoint blockade (74, 75), and total neoadjuvant regimens. These converge on ordered timing but do not yet unify all three axes on one time axis specific to cold tumors. The central hero schema, termed TMEp-CI-M and presented as one hypothesis-generating exploration among several that will be needed, arranges three axes along one time axis aligned to the open immunologic window. Tumor microenvironment priming (radiotherapy at its core, optionally reinforced by vascular-normalizing anti-angiogenics or metronomic chemotherapy) supplies antigen and access and diversifies the intratumoral T-cell-receptor repertoire; checkpoint inhibition is delivered into the open window, with a decision rule that blockade depth (monotherapy versus dual or bispecific) matches the breadth of priming, reflecting that anti-PD-1/PD-L1 reverses exhaustion, whereas anti-CTLA-4 acts on a distinct substrate (mobilizing ICOS-positive Th1-like CD4 cells and, in preclinical models, depleting intratumoral regulatory T cells); and microbiome modulation runs continuously beneath both as a background layer setting systemic permissiveness. Order and interval, not simultaneous combination, determine whether a cold tumor is ignited, and the three axes address non-overlapping deficits. The lower band states a concrete, testable schedule and its falsifiable predictions on a shared time axis: microbiome conditioning begins first and continues throughout (initiated one to several weeks before local therapy, with antibiotics avoided in the pre-checkpoint window (19)); hypofractionated radiotherapy (8 Gy × 3) then opens the local window; and checkpoint inhibition begins within roughly 1 week of the last radiotherapy fraction, matched in depth to priming breadth. Each interval is a parameter to be optimized rather than a settled value, and each carries a falsifiable prediction testable in biomarker-driven trials: microbiome-first versus microbiome-concurrent arms, day-1 co-dosing versus delayed blockade, and dual versus depth-matched blockade in narrowly primed tumors. The schema is hypothesis-generating and is not presented as validated or as superior to standard care. Created in https://BioRender.com.

Evidentiary status of the observations that follow and of **Table 4**: The cross-tumor experience here comes from small single-arm cohorts, retrospective series, and individual case reports and is, therefore, exposed to selection bias, to publication and reporting bias, and to confounding by concomitant therapy, prior lines, and disease burden at entry. What this material can establish is that a time-sequenced regimen is deliverable and which sequences deserve randomizing first. That is a generative role rather than a confirmatory one: these reports are not evidence of efficacy; they do not establish superiority over standard care.

An operational version of this logic has been applied clinically across six cold-tumor types in a single-institution experience, evolving from a fixed multimodal regimen (originally termed BRICS) into the generalized three-axis arrangement described above (**Table 4**): PD-L1-negative NSCLC (110), mismatch-repair-proficient colorectal cancer (111), pancreatic neuroendocrine carcinoma (112), locally advanced pancreatic cancer (113), small-cell lung cancer (114), and unresectable HCC (115). The earliest and most illustrative experience was a pancreatic neuroendocrine carcinoma that was mismatch-repair-proficient, PD-L1-negative, and highly proliferative (Ki67 80%)—a phenotype (CPS 0, pMMR) predicting minimal checkpoint benefit; after multi-site radiotherapy followed by PD-1 blockade with continuous probiotic support, the patient achieved a complete response and has remained without evidence of disease for over 7 years (112). The remaining cases, detailed in **Table 4**, include a prospective single-arm HCC study in which SBRT (8 Gy × 3) was used to expose tumor antigens before toripalimab plus anlotinib (115) and a brainstem-metastatic small-cell lung cancer in which a priming phase preceded a PD-1/CTLA-4 bispecific with concurrent probiotics (114).

**Table 4:** Cross-tumor proof-of-concept experience with a three-axis, time-sequenced regimen.

Tumor type (cold marker)	Priming → checkpoint → microbiome mapping	*n*	Design	Key outcome (follow-up)	Grade ≥3 AE	Ref.
PD-L1-negative NSCLC (EGFR/ALK WT)	SBRT 24 Gy/3 + low-dose chemo → PD-1 → probiotics	23	Retrospective	ORR/DCR 95.7%; mPFS 16 mo, mOS 32.7 mo	None (all grade 1–2)	(110)
pMMR/MSS colorectal, refractory	SBRT + low-dose chemo → PD-1 → high-dose probiotics	27	Retrospective salvage	ORR 33.3%, DCR 88.9%; mPFS 7.2 mo, mOS 12.3 mo	22.2% (neutropenia 14.8%)	(111)
Neuroendocrine carcinoma (Ki67 80%, CPS 0, pMMR)	Multi-site RT → PD-1 → continuous probiotics	1	Case	CR; NED >7 years	NR	(112)
Locally advanced pancreatic (pMMR, CPS 30)	SBRT 24 Gy/3 + anlotinib + low-dose chemo → toripalimab → *Bifidobacterium*	1	Case	CR; markers normalized (5 mo)	None reported	(113)
SCLC, brainstem metastasis (PD-L1-neg ES)	SBRT + low-dose etoposide + anlotinib → PD-1/CTLA-4 bispecific → probiotics	1	Case	NED >20 mo (ProGRP 1,803 → 24 pg/mL)	None (grade 1 only)	(114)
Unresectable HCC (BCLC B/C)	SBRT 8 Gy×3 → toripalimab + anlotinib	20	Prospective single-arm	ORR 15%, DCR 50%, mPFS 7.4 mo; 1 pathological CR	NR (any-grade TRAE 70%)	(115)

Single-institution, cross-tumor proof-of-concept experience with a three-axis, time-sequenced regimen (priming → checkpoint inhibition, with microbiome modulation sustained throughout). These experiences are hypothesis-generating: they are uncontrolled, small, single-arm, retrospective, or single-patient, and do not establish superiority over standard care. All efficacy figures were checked against the primary publications. The “→” symbol in the mapping column denotes the intended temporal order; in several cases, the microbiome component was given concurrently rather than as pre-conditioning, so the series supports deliverability but not the specific timing claim (see Section 10.2).

AE, adverse event; CPS, combined positive score; CR, complete response; DCR, disease control rate; ES, extensive-stage; HCC, hepatocellular carcinoma; mo, months; mOS/mPFS, median overall/progression-free survival; MSS, microsatellite-stable; NED, no evidence of disease; NR, not reported; NSCLC, non-small cell lung cancer; ORR, objective response rate; pMMR, mismatch-repair-proficient; ProGRP, pro-gastrin-releasing peptide; SBRT, stereotactic body radiotherapy; SCLC, small-cell lung cancer; TRAE, treatment-related adverse event; WT, wild type.

These observations must be read with strict calibration. The series comprises small, single-arm, retrospective, or single-patient experiences (*n* ≈ 20–27 in the cohort studies), without randomized controls, and is subject to selection and publication bias; the outcomes are hypothesis-generating signals of feasibility, not evidence that this or any specific arrangement is superior to standard care. Critically, these cases also do not test the framework’s own timing claims: the microbiome component, in particular, was delivered concurrently rather than as the early, sustained pre-conditioning layer the schema proposes, so the series supports the deliverability of a multimodal, prime-then-inhibit stack but not the specific temporal ordering that is its central hypothesis. Their value is to demonstrate that a time-sequenced, multi-axis approach is deliverable across diverse cold tumors and to motivate the controlled trials that would actually test it.

### 10.3 A testable sequencing schema

To be useful, any framework built on timing must state its intervals concretely enough to be proven wrong. Synthesizing the evidence above, a first-approximation schema for a cold solid tumor would run as follows. Microbiome conditioning begins first and continues throughout, initiated one to several weeks before any local therapy so that a favorable systemic set-point has time to form (Section 8) and antibiotic exposure is avoided in the pre-checkpoint window (19). Priming then opens the local window—hypofractionated radiotherapy (8 Gy × 3), optionally reinforced by a vascular-normalizing anti-angiogenic and metronomic chemotherapy over the same interval. Checkpoint inhibition is delivered into the primed window, beginning within roughly 1 week of the last radiotherapy fraction, the interval used in PEMBRO-RT (35), and matched in depth (mono- versus dual or bispecific blockade) to the expected breadth of the primed repertoire. Every element of this schema is a hypothesis: the pre-conditioning lead time, the priming-to-checkpoint interval, and the depth-matching rule are parameters to be optimized, not settled values. Each also carries a falsifiable prediction: if the pre-conditioning lead time were irrelevant, microbiome-first and microbiome-concurrent arms would perform identically; if the priming-to-checkpoint interval were unimportant, day-1 co-dosing would match delayed blockade; and if depth-matching were spurious, dual blockade would add only toxicity in narrowly primed tumors. Stating them explicitly is the point—each becomes a variable that a biomarker-driven trial can test and falsify, which is precisely what the addition paradigm, lacking a time axis, cannot offer.

## 11 Challenges, controversies, and future directions

Translating a timing-based approach from principle to practice requires resolving several open questions and demands candor about where the evidence is strong and where it is not.

The most important limitation is an asymmetry in the evidence. The directional signal that sequential beats concurrent is, at present, clinical only for radiotherapy plus checkpoint blockade (16), reinforced by the neoadjuvant-versus-adjuvant trials in which the same agent changes outcome with timing (37, 38). Extending the timing principle to the anti-angiogenic and microbiome axes rests on mechanistic and preclinical inference rather than on comparative human sequencing data and is, therefore, the central untested extrapolation of any integrated schema. Whether microbiome modulation must precede rather than merely accompany checkpoint blockade, in particular, has not been tested directly since most FMT and probiotic trials use concurrent designs.

The foundational sequencing question is itself unsettled and moving: as noted (Section 6.2), the primary clinical evidence is a network meta-analysis rather than a head-to-head sequencing trial, with confirmatory randomized trials ongoing, so any application of a timing framework must be date-stamped to the current state of that evidence (16). There are also genuine counter-considerations: in some settings, concurrent delivery may capture synergies that separation forfeits, and the optimal interval may differ by histology, by primer, and by the depth of checkpoint blockade used. The assumption that priming must always strictly precede blockade may also not hold universally: where a modest pre-existing infiltrate exists, early checkpoint blockade could protect newly primed cells from immediate re-exhaustion, making a briefly overlapping schedule preferable to complete separation. Sequencing must additionally be weighed against acquired resistance, which can close a window that timing had opened and which remains poorly understood mechanistically (25). A timing framework does not by itself solve acquired resistance, but it reframes part of it as a scheduling problem: if a window closes because adaptive counter-regulation outpaces a delayed intervention, the remedy may be tighter coupling of the readout to the response rather than another drug.

### 11.1 When concurrent administration may outperform sequential delivery

The directional signal assembled in Sections 4 and 6.2 should not be read as a universal rule, and the randomized evidence contains genuine counter-examples. The clearest is KEYNOTE-A18 in high-risk locally advanced cervical cancer. Pembrolizumab, given concurrently with chemoradiotherapy and then continued, improved progression-free survival over chemoradiotherapy alone (24-month progression-free survival 68% versus 57%, hazard ratio 0.70, *p* = 0.0020). Overall survival subsequently improved as well (36-month overall survival 82.6% versus 74.8%, hazard ratio 0.67, *p* = 0.0040) (14, 15). Here concurrency proved definitive rather than merely tolerable, at the cost of roughly doubled immune-mediated adverse events (39% versus 17%) (15). PACIFIC-2 supplies the mirror image and shows that the question is genuinely empirical. In unresectable stage III NSCLC, durvalumab started simultaneously with concurrent chemoradiotherapy produced no gain over chemoradiotherapy alone. Progression-free survival was unchanged (hazard ratio 0.85, 95% CI 0.65–1.12, *P* = .247), as was overall survival (hazard ratio 1.03, 95% CI 0.78–1.39, *P* = .823). Adverse events leading to discontinuation were more frequent (25.6% versus 12.0%), so consolidation after chemoradiotherapy remains the standard (116). The two trials share a design logic yet diverge in result, which argues that the optimal schedule is disease-specific rather than dictated by a general principle.

Four situations plausibly favor concurrency, and each is testable. First, the primer may itself be curative-intent and undeferrable without compromising local control, as in definitive chemoradiotherapy for cervical cancer. The checkpoint inhibitor must then either accompany it or wait out a protracted course, and waiting risks losing the antigen-rich interval altogether. Second, when a modest T-cell infiltrate already exists, early blockade may protect newly primed cells from immediate re-exhaustion rather than arriving before there is anything to disinhibit. Read strictly, the preclinical finding that PD-L1 blockade must fall within days of fractionated radiotherapy to overcome radiation-induced resistance (54) argues for tight overlap rather than for separation. Third, when the primer’s window is very short, separation can forfeit it. The vascular-normalization window (Section 5.2) is measured in days, so anti-angiogenic-plus-ICI regimens such as APOLLO may be capturing a real synergy that a deliberate delay would miss (78). Fourth, in rapidly progressing disease, the competing risk is progression during the priming interval, which is the practical objection to any protracted sequence in cold, high-burden tumors. Concurrency also has a logistical advantage that a timing framework must concede in that it compresses the treatment course and requires less cross-disciplinary coordination.

Read together, these observations refine rather than refute the review’s thesis. What the evidence supports is not that sequential always beats concurrent but that schedule is a modifiable determinant of benefit whose optimum depends on the primer, the disease, and the pre-existing immune state. In tumors with some baseline immune engagement and a non-deferrable curative primer, concurrency may be preferable. In immune-desert tumors, the primer’s purpose is to manufacture an infiltrate that does not yet exist, and there prime-then-inhibit remains the better-supported hypothesis. Adjudicating between the two demands trials that randomize the interval while holding agents constant, which is the experiment proposed below, rather than further single-schedule combinations.

Methodologically, no trial has yet evaluated the full stack (priming, checkpoint blockade, and microbiome conditioning) together. Doing so with conventional two-arm designs is impractical because the number of order-and-interval permutations is large. Adaptive, biomarker-driven master protocols, which can compare multiple sequencing schedules against a common control and drop inferior arms as data accrue, are far better suited to isolating the contribution of timing rather than of added agents (117). Such designs will depend on the dynamic readouts of window state discussed in Section 9, none of which is yet prospectively validated for sequencing, to assign the next intervention to the moment the biology is ready for it rather than to a fixed calendar.

Practical deliverability is a further often-neglected constraint. A time-sequenced, multi-axis regimen is logistically heavier than a single infusion: it requires coordination across radiation oncology, medical oncology, and, if the microbiome layer is used, microbiology and infectious-disease services sustained over weeks. In frail, heavily pre-treated patients who typify cold disease, the tolerability advantage of separating toxicities in time (Section 6.2) must be weighed against the burden of a longer, more complex course and the risk that progression during a protracted priming phase forecloses the window before checkpoint blockade is ever delivered. Health-economic and access considerations follow directly: fecal microbiota transplantation, hypofractionated radiotherapy, and bispecific antibodies differ enormously in cost, infrastructure, and availability, so a schema that is optimal biologically may be undeliverable in many settings. These are not reasons to abandon sequencing but parameters that trials and any eventual guideline must incorporate explicitly, with an emphasis on identifying the simplest sequence that captures most of the benefit rather than the most elaborate one.

Set against these burdens is an economic argument that distinguishes a timing strategy from most other routes to improve outcomes in cold tumors. Every agent in the sequences discussed here is already approved and already in use. A schedule change, therefore, requires no new molecule, no first-in-human program, and no new manufacturing. Its incremental cost over the same agents given concurrently is close to the cost of the extra visits and monitoring that separation entails. That compares favorably with developing a novel immunotherapeutic. There attrition is steep, and development costs are measured in the billions and are ultimately reflected in the price. The median gain delivered by a successful new agent in cold disease has so far been modest. Repositioning and re-scheduling existing drugs is attractive for exactly this reason: established pharmacology, known toxicity profiles, and existing supply chains shorten the path to evaluation and lower its cost (118). The same logic underlies the interest in low-dose metronomic scheduling of old cytotoxics as immune modulators, where cost is a stated advantage rather than an afterthought (65, 76, 119). Two qualifications keep this from being an unmitigated claim. A longer, multi-disciplinary course consumes more clinician and facility time even when drug costs are unchanged. The microbiome axis, if it uses fecal microbiota transplantation or a manufactured consortium, adds a genuinely new cost and infrastructure requirement rather than re-using an existing one. The economic case is, therefore, strongest for the radiotherapy and anti-angiogenic axes, where the components are generic or already reimbursed. It argues for a formal cost-effectiveness analysis alongside any randomized schedule trial rather than in place of one. It also reinforces the point made above—that the objective should be the simplest sequence capturing most of the benefit.

None of this diminishes the near-term testability of the core claim. The single most consequential experiment is also among the simplest: within an established priming-plus-checkpoint regimen, randomize the interval, concurrent versus a defined delay, while holding the agents and doses constant, exactly as the neoadjuvant-versus-adjuvant trials have already done for surgery-linked immunotherapy (37, 38). A positive result would establish timing as an independent, modifiable determinant of benefit; a negative one would bound the hypothesis. Either outcome would advance the field more than another concurrent doublet.

### 11.2 Statistical considerations for an adaptive sequencing trial

Several intervals and several priming modalities are candidates at once, so a fixed two-arm design is an inefficient way to ask this question. The appropriate vehicle is a Bayesian adaptive platform in which schedule arms share a common control (117, 120). Three statistical issues then dominate. The first is interim, biomarker-driven arm dropping. Response-adaptive randomization and pre-specified futility rules allow an interval arm to be abandoned early and biomarker-defined strata to be enriched or closed as data accumulate. Three requirements follow. The biomarker used for adaptation must be measurable in real time and locked before the first interim. Futility must be expressed as a pre-stated posterior probability rather than decided case by case. The operating characteristics, meaning the probability of dropping a truly effective arm and of retaining an ineffective one, must be established by simulation before enrollment (120). Comparing multiple arms against one shared control induces multiplicity that has to be handled explicitly rather than ignored. A shared control inflates the family-wise error rate across arms and also correlates the arm-versus-control comparisons (121). The dynamic readouts of Section 9 are the natural adaptation variables, but none is yet validated for that role. A first-generation trial should, therefore, adapt on clinical endpoints while collecting the candidate biomarkers prospectively for later qualification.

The second issue is sample size, and it is the reason interval questions have gone untested. A schedule trial does not ask whether a drug works, but whether the same drugs work better in a different order. The expected effect is, therefore, smaller than the effect of adding an agent, and the required sample is larger. The available anchor for the magnitude is the network meta-analysis, in which sequential delivery improved progression-free survival over concurrent delivery with a hazard ratio of 0.81 (16). Detecting a hazard ratio of that size with 80% power at a two-sided alpha of 0.05 requires on the order of 380 events. In a population with the event rates typical of advanced cold disease, that means roughly 450 to 500 patients randomized one-to-one. A hazard ratio of 0.75 would need about 250 events. Both are well beyond the size of any trial that has so far compared schedules, and both assume that the schedule effect is uniform across histologies, which Section 2 argues it is not. Three consequences follow. A single-histology, single-center interval trial is almost certainly underpowered and should not be attempted as a definitive study. A platform with a shared control arm recovers much of the lost efficiency and is the realistic route to an adequately powered comparison (117, 120). The appropriate first step is a randomized phase II with a mechanistic co-primary endpoint, powered for the immunological change rather than for survival. A schedule that does not move the intratumoral effector wave is unlikely to move survival and can be abandoned cheaply.

The third issue is the choice of primary endpoint, where a timing trial has a specific difficulty. Objective response rate suits an early schedule-finding stage because it reads out quickly and is sensitive to whether the window was exploited at all. It is nonetheless a poor guide to the durability that the mechanistic argument predicts. In cold tumors, a low response rate also leaves little room to discriminate between schedules. Overall survival remains the endpoint that matters and is the appropriate primary endpoint for the confirmatory stage. It is also the most confounded by subsequent lines of therapy, and the interval effect can be diluted by crossover. Durable clinical benefit, pre-specified as response or stable disease sustained beyond a fixed landmark, best matches the hypothesis. The claim being tested is that correct timing converts transient into sustained disinhibition. It should be defined by landmark rather than by investigator judgment and reported with the landmark stated. A defensible structure is, therefore, objective response rate plus a mechanistic co-primary endpoint at the schedule-finding stage, with durable clinical benefit as the primary endpoint of the randomized comparison. Overall survival becomes a pre-specified key secondary endpoint, with a hierarchical testing order fixed in advance so that the survival analysis is interpretable rather than exploratory.

Finally, a timing-based program should be positioned honestly against the broader immunotherapy agenda. The general case that temporal dynamics matter has been made across modalities and diseases (44), and the wider effort to rationalize checkpoint combinations continues in parallel (34). Adjacent literatures are converging on the same scheduling question from other directions. Delivery technology determines where and for how long an immunotherapeutic is present and, therefore, what a nominal schedule actually achieves at the tumor (79). Computational approaches are being applied to chemo-immunotherapy scheduling and to nanocarrier-based design, where dose-and-interval permutations exceed what empirical testing can cover (80, 81). Engineered bacterial immunotherapy shows that the microbial axis can be programed for controlled, time-resolved activity inside the tumor rather than treated as a fixed background. An IL-10R hysteresis circuit there permits phagocytosis evasion and later revitalization of antitumor immunity (99). These are complementary rather than confirmatory: none of them randomizes the interval between priming and checkpoint blockade, which remains the untested core of the present argument. A cold-tumor-specific, mechanism-anchored, falsifiable schema is narrower and more operational than either and complementary to both—the general principle motivates the specific strategies, and the specific strategies supply the testable predictions that turn the principle into trials.

## 12 Conclusion

For immunologically cold tumors, the accumulating evidence argues that the field is shifting from addition (combining more agents, usually at once) toward timing, in which the order and interval of interventions determine success. The temporal biology of priming, vascular normalization, adaptive resistance, and microbiome remodeling defines a shared window of opportunity, and the strongest available clinical signals, from radiotherapy plus checkpoint blockade and from neoadjuvant-versus-adjuvant dosing, suggest that aligning checkpoint inhibition to that window can outperform simultaneous stacking. Several groups are now translating this principle into integrated, time-sequenced strategies; the three-axis schema described here is one concrete, falsifiable instantiation—prime the microenvironment to open the window, deliver checkpoint inhibition while it is open, and condition the host microbiome throughout—illustrated by hypothesis-generating cross-tumor experience rather than validated as a protocol. Its purpose, and that of the field’s parallel efforts, is to convert the intuition that the order of interventions is itself therapeutic into a structured agenda (defined intervals, full-stack adaptive trials, and dynamic biomarkers of window state) for the next generation of cold-tumor immunotherapy. If even a fraction of the non-responding majority in cold disease is failing not for want of the right agents but for want of the right order, then re-sequencing what we already have is among the highest-yield and lowest-cost hypotheses in immuno-oncology—and one that can be tested now.
